# The structure and function of an RNA polymerase interaction domain in the PcrA/UvrD helicase

**DOI:** 10.1093/nar/gkx074

**Published:** 2017-02-04

**Authors:** Kelly Sanders, Chia-Liang Lin, Abigail J. Smith, Nora Cronin, Gemma Fisher, Vasileios Eftychidis, Peter McGlynn, Nigel J. Savery, Dale B. Wigley, Mark S. Dillingham

**Affiliations:** 1 DNA:Protein Interactions Unit, School of Biochemistry, Biomedical Sciences Building, University of Bristol, Bristol BS8 1TD, UK; 2 Institute of Cancer Research, Chester Beatty Laboratories, 237 Fulham Road, London SW3 6JB, UK and Section of Structural Biology, Department of Medicine, Imperial College London, South Kensington Campus, London SW7 2AZ, UK; 3 Department of Biology, University of York, Wentworth Way, York YO10 5DD, UK

## Abstract

The PcrA/UvrD helicase functions in multiple pathways that promote bacterial genome stability including the suppression of conflicts between replication and transcription and facilitating the repair of transcribed DNA. The reported ability of PcrA/UvrD to bind and backtrack RNA polymerase (1,2) might be relevant to these functions, but the structural basis for this activity is poorly understood. In this work, we define a minimal RNA polymerase interaction domain in PcrA, and report its crystal structure at 1.5 Å resolution. The domain adopts a Tudor-like fold that is similar to other RNA polymerase interaction domains, including that of the prototype transcription-repair coupling factor Mfd. Removal or mutation of the interaction domain reduces the ability of PcrA/UvrD to interact with and to remodel RNA polymerase complexes *in vitro*. The implications of this work for our understanding of the role of PcrA/UvrD at the interface of DNA replication, transcription and repair are discussed.

## INTRODUCTION

Helicases are ubiquitous, abundant and diverse enzymes playing a wide variety of different roles in cellular nucleic acid metabolism (3). Several superfamilies (SF) of these enzymes have been described on the basis of primary structure and SFI and II, which are non-hexameric helicases, are by far the largest groups (4). Bacterial cells typically encode several SFI enzymes that function in different genome replication, maintenance and expression pathways (5). Structural studies have shown that SFI enzymes share highly conserved core helicase domains responsible for ATP-dependent DNA translocation and unwinding, and that their targeting to different pathways is often achieved via the modular addition of different specificity domains, either flanking or inserted within the core helicase domains (6).

An interesting example is provided by the UvrD helicase (also annotated Helicase II, or PcrA in many Gram positive bacteria including *Bacillus subtilis*) which has been implicated in nucleotide excision repair (NER), mismatch repair, homologous recombination and rolling circle replication mechanisms (7–13). This multi-functionality is reflected in the ability of UvrD/PcrA to interact physically and functionally with many different partner proteins including UvrB, MutL, MutS, RecA and RepC/D (13–20). We and others have recently shown that PcrA/UvrD also interacts with RNA polymerase (RNAP) (1,14,21), and this interaction could be important for the UvrD-dependent backtracking of stalled RNA polymerase (1). It was suggested that this activity helps to recruit the NER machinery to sites of UV damage, acting as an alternative pathway of transcription-coupled repair (TCR) in addition to the well-characterized Mfd pathway (for reviews see (22–25)). This ability of UvrD to remodel RNAP-DNA complexes might also be relevant to the ability of PcrA/UvrD to suppress conflicts between replication and transcription, a property it shares with the very closely related helicase Rep (although Rep does not interact with RNAP) (26–28). The extreme C-terminal region of PcrA/UvrD is important for interaction with both RNAP (14,29) and UvrB (30). However, despite its apparent role as a protein interaction hub that targets the helicase to physiological substrates, there is no clear phenotype established for removal of the C-terminal domain (CTD), and its structure has never been resolved. In thirteen structures of the PcrA/UvrD protein from various organisms, this region of the protein was either removed to aid crystallization or is disordered in the final model (31–36) (Supplementary Figure S1).

In this report, we define a small folded RNA polymerase interaction domain in *Geobacillus stearothermophilus* PcrA and have solved its structure at high resolution. Based on its similarity to other Tudor-like domains, we identify conserved residues on its surface that are likely to be directly involved in the interaction with RNAP. Mutation of these residues, or the complete removal of the interaction domain, substantially reduces the ability of PcrA/UvrD to bind to RNAP and to remodel transcription elongation complexes *in vitro*.

## MATERIALS AND METHODS

### Protein expression and purification

His-tagged *Escherichia coli* RNAP holoenzyme was purified as described (37). GreB protein was a gift from Terence Strick. Purified ParB protein was a gift from James Taylor. Biotinylated BSA protein was purchased from ThermoScientific. UvrD and UvrD^K708A^ were purified from BL21(DE3) cells transformed with pETDUET-UvrD (15) or pETDUET-UvrD^K708A^, which was made from pETDUET-UvrD by site directed mutagenesis. Cells were grown at 37°C to an *A*_600_ of ∼0.5 before being used to inoculate 1 l of LB + 100 μg/ml ampicillin to an *A*_600_ of ∼0.025. When cells reached an *A*_600_ of 0.2 they were transferred to 18°C and when the *A*_600_ reached ∼0.5, 1 mM IPTG was added to induce protein expression. Cultures were then grown overnight at 18°C and the cells were harvested by centrifugation at 4°C. The cell pellet was resuspended in 20 ml of lysis buffer (20 mM Tris–HCl pH 8.3, 10% (v/v) glycerol, 200 mM NaCl, 5 mM EDTA pH 8.0, 0.5 mM EGTA pH 8.0, 1 mM DTT) containing 4 mg of lysozyme and incubated on ice for 30 min. 250 μl 4% sodium deoxycholate was added and cells were then incubated on ice for another 30 min. To increase the UvrD solubility, NaCl concentration was increased to ∼450 mM by adding 1.2 ml 5 M NaCl and stirring for 15 min at 4°C. The cells were lysed by sonication and the soluble fraction was recovered by centrifugation. In order to precipitate the UvrD, saturated ammonium sulphate was added gradually to the supernatant until 30% saturation was reached. The protein was allowed to precipitate for 1 h in an ice waterbath and was recovered by centrifugation at 4000 rpm for 30 min. The pellet was resuspended in buffer (20 mM Tris–HCl pH 8.3, 20% (v/v) glycerol, 400 mM NaCl, 2 mM EDTA pH 8.0, 0.5 mM EGTA pH 8.0, 1 mM DTT) and was slowly diluted in buffer A (20 mM Tris–HCl pH 8.3, 20% (v/v) glycerol, 1 mM EDTA pH 8.0, 0.5 mM EGTA pH 8.0 and 1 mM DTT) until the salt concentration was approximately 200 mM NaCl (or 100 mM for the UvrD^K708A^ mutant). This was then loaded onto a 5 ml Heparin column (GE) on an ÄKTA FPLC. The protein was eluted from the column using a NaCl gradient in buffer A. Fractions containing the protein of interest were combined and diluted in buffer A until the salt concentration was approximately 200 mM NaCl (or 100 mM for the UvrD^K708A^ mutant). Protein was loaded onto a 1 ml MonoQ column (GE) on an ÄKTA FPLC. The protein was eluted using a NaCl gradient in buffer A. Fractions containing only the protein of interest were dialysed at 4°C overnight against storage buffer (20 mM Tris–HCl pH 8.3, 200 mM KCl, 1 mM EDTA pH 8.0, 2 mM DTT, 20% (v/v) glycerol). UvrDΔC was purified from BL21DE3 cells transformed with pETDUET-UvrD_1–647_ as described (15).

Biotinylated PcrA and biotinylated PcrA-Ct (including residues 653–724 of the native protein) were produced using vectors and purification protocols that have been described previously (14). An equivalent vector for expression of biotinylated PcrA-sCt was created by deleting a short region of the PcrA-Ct construct. This yielded a vector for overexpression of the extreme C-terminal region of PcrA (residues 673–724) fused to an N-terminal AviTag sequence (MSG LND IFE AQK* IEW HEG GG; the asterisk indicates the biotinylated lysine). This protein was overexpressed and purified using the same method as for the PcrA-Ct construct. Constructs for the expression of the histidine-tagged PcrA C-terminus were produced by cloning synthetic DNA (Invitrogen) into the pET47b vector (Novagen). The PcrA-Ct construct expresses a protein with an N-terminal hexa-histidine tag fused via a 3C cleavable linker to residues 653–724 of *Geobacillus stearothermophilus* PcrA. The sequence of the tag is MAH HHH HHS AAL EVL FQG *PGG G where the asterisk indicates the position of 3C cleavage. The PcrA-sCt construct only codes for residues 673–724 of the native PcrA protein, but is otherwise equivalent to PcrA-Ct. Point mutations were made in all of the above vectors using the QuikChange II kit (Invitrogen) and the constructs were verified by DNA sequencing (Sequencing service, University of Dundee). His-tagged PcrA-Ct and his-tagged PcrA-sCt were overexpressed in BL21(DE3) with appropriate antibiotics and harvested using the same protocol as for full-length PcrA (14). Following sonication, the proteins were bound to a 5 ml HisTrap column (GE Healthcare) in a buffer containing 50 mM Tris–Cl, pH7.5 and 200 mM NaCl, and eluted over a 20–500 mM imidazole gradient. Where appropriate, the his-tag was removed with HRV 3C protease overnight at 4°C (ThermoScientific, manufacturer's instructions) while dialysing against a buffer containing 50 mM Tris–Cl, pH7.5, 1 mM EDTA, 200 mM NaCl and 20 mM imidazole. The cleaved protein was re-passed over the HisTrap column to remove HRV 3C protease contamination and PcrA S-Ct was collected in the flow-through. The cleaved (or uncleaved) protein was finally purified using a Superdex75 gel filtration column (GE Healthcare) in a buffer containing 50 mM Tris–Cl, pH7.5, 1 mM EDTA, 1 mM DTT and 200 mM NaCl. Peak fractions were pooled and concentrated using a 3 kDa cut-off spin concentration device. Where appropriate, removal of the tag was verified by separation using a 10–20% Tris–tricine gel by comparison with the his-tagged protein. The concentration of protein was determined by spectrophotometry using a theoretical extinction coefficient of 11 000 M^−1^ cm^−1^. The protein was snap frozen and stored at –80°C in a buffer containing 50 mM Tris–Cl, pH7.5, 1 mM EDTA, 200 mM NaCl, 1 mM DTT and 10% glycerol.

### Crystallization, structure determination and structure analysis

The PcrA-sCt protein (with the his-tagged removed) was concentrated to 20 mg/ml in a buffer of 50 mM Tris-Cl (pH 7.5), 1 mM EDTA, 200 mM NaCl and 10% glycerol. Crystals were grown using the sitting-drop vapor diffusion method at 18°C by mixing 0.2 μl protein solution with 0.2 μl precipitant solution containing 3.5 M sodium formate pH 7.0. Crystals were harvested and frozen using the precipitant solution. A mercury derivative was prepared by soaking the crystals for 4 h in cryosolution (3.5 M sodium formate pH 7.0) containing 10 mM ethyl mercury phosphate, and then freezing. X-ray diffraction data were collected at 100 K using a Rigaku FR-X X-ray generator and PILATUS 300K detector. The data were processed and scaled using the HKL3000R program (38). A single mercury site was found by direct methods in SHELXD (39). Heavy-atom refinement and phasing (using SIRAS) were performed using SHARP (40). Solvent flipping and density modification were performed using SOLOMON (41) and Parrot (42), respectively. An initial model was built automatically using Buccaneer (43) and the final structure model was manually rebuilt using Coot (44) with refinement in Refmac (45) and Phenix (46). Diffraction data and refinement statistics are listed in Table 1, and the co-ordinates of the structure have been deposited at the PDB under ID code 5DMA. Figures showing the conservation of residues in PcrA mapped onto the structure were created using ConSurf (47) and PyMOL. The multiple sequence alignment was based on 150 unique sequences that were the most similar to the *G. stearothermophilus* PcrA C-terminus using the ConSurf default input settings.

**Table 1.** X-ray data collection and refinement statistics for PcrA-sCt

	Hg	Native
**Data collection statistics**		
Wavelength (Å)	1.54	1.54
Space group	*P*3_2_21	*P*3_2_21
Cell dimensions (*a, b, c*) (Å)	50.4, 50.4, 40.4	50.5, 50.5, 40.2
Resolution (Å)	50–1.7 (1.8–1.7)^a^	50–1.5 (1.6–1.5)^a^
Observed/unique reflections	41 952/6980	92 776/9252
Data redundancy	6.0 (3.0)	10.0 (5.1)
Completeness (%)	99.3 (98.7)	99.9 (98.9)
Rsym (%)	7.5 (15.8)	3.4 (18.5)
*I*/σ(*I*)	33.6 (8.1)	61.6 (7.7)
**Refinement statistics**		
Resolution range (Å)		30.0–1.5
Reflections (work/test)		9167/438
R_work_/R_free_ (%)		19.5/22.2
Number of atoms (protein/water)		407/65
Average B-factor (protein/solvent) (Å^2^)		15.2/25.5
RMSD in bond length (Å)/bond angle (°)		0.007/1.193

^a^Values in parentheses refer to the highest resolution shell.

### Pulldown assays

Pulldown assays were performed as in previous work (14) using either streptavidin-coated magnetic beads (New England Biolabs) for biotin-tagged bait proteins or substituting Ni^2+^-NTA magnetic beads (New England Biolabs) for his-tagged bait proteins. Briefly, DNA/RNA-depleted extracts of *B. subtilis* 168 were produced as described previously (14). *B. subtilis* was chosen as the source for the bait proteins because the PcrA from this organism is highly similar (85%) to its orthologue from *G. stearothermophilus*, and because the annotated proteome is important for the proteomics analysis (below). Nevertheless, it should be noted that our pulldown experiments may be susceptible to false negatives because of the different *Bacillaceae* species used for bait and prey proteins. The prepared extracts were then used as the prey in pulldown experiments. Purified bait proteins were incubated at near saturating concentrations with magnetic beads to allow binding. The baited beads were washed and added to *B. subtilis* cell extracts to allow binding with partner proteins. The beads were separated from the cell extract and washed before bait and prey proteins were harvested from the beads by boiling in SDS-PAGE sample buffer. The pulldown experiments were analysed by SDS-PAGE followed by either western blotting with an anti-RNAP β antibody (8RB13, Abcam (48)), or by mass spectrometry. Details of sample preparation for mass spectrometry can be found in the Supplementary Information.

### Construction of templates for *in vitro* transcription assays

Linear DNA templates for *in vitro* transcription reactions were constructed by PCR amplification from pSRT7A1 (49) using Pfu DNA polymerase. Templates containing the T7A1 promoter were amplified using the upstream primer 5΄-ACCTGACGTCTAAGAAACC-3΄ and the downstream primer 5΄-ATTACTGGAGGGGATGGGG-3΄ to produce a 236 bp product. On this template transcription can be stalled by nucleotide starvation at +20 by omitting UTP. Transcription can also be chased to the template end to produce a transcript of 60 nt. Linear biotinylated DNA template was made in a similar manner using a 5΄-biotinylated upstream primer and DNA templates were purified using the QIAEX II DNA extraction kit (Qiagen). Plasmid pSRTB8B3+500 was created by inserting a 504 bp fragment amplified from the *E. coli rpoB* gene between the NcoI and XhoI sites located between the T7A1 promoter and the tandemly repeated BbvCI sites of pSRTB8B3 (50). On the resulting template transcription from the T7 promoter can be stalled at +20 by UTP starvation, or allowed to run to a downstream terminator to produce a 764 nt transcript. A biotinylated closed-circular plasmid template carrying a biotin-dT at position +585 on the transcribed strand was generated by annealing a biotin-dT-containing oligonucleotide into BbvCI-nicked plasmid pSRTB8B3+500, as described in (50).

### *In vitro* transcription time course and transcript release assays

For time course assays, transcription initiation complexes were formed by incubating 20 nM *E. coli* RNA polymerase holoenzyme with 2 ng/μl DNA template for 5 min at 37°C in repair buffer (40 mM HEPES, pH 8.0, 100 mM KCl, 8 mM MgCl_2_, 4% glycerol (v/v), 5 mM DTT, 100 μg/ml BSA). Transcription elongation complexes stalled at +20 were then formed by nucleotide starvation: the preformed transcription initiation complexes were mixed with an equal volume of NTP stall mix in repair buffer (final concentrations 100 μM ApU, 10 μM ATP, 10 μM GTP, 2 μM CTP, 0.5 μCi/μl [α-^32^P] CTP) and incubated for 5 min at 37°C. Aliquots of the stalled elongation complexes were incubated with UvrD or its derivatives at the concentrations indicated for 5 min at 37°C. Transcription elongation was then allowed to continue for 5 min at 37°C by adding a ‘chase’ of 100 μM NTPs, together with 10 μg/ml rifamipicin to ensure that only a single round of transcription took place. Reactions were stopped with an equal volume of urea stop mix (7 M urea, 10 mM EDTA, 1% SDS, 2× TBE, 0.05% bromophenol blue, 0.05% xylene cyanol). Samples were heated for 3 min at 90°C and resolved on a 15% polyacrylamide/7 M urea denaturing gel. Gels were analyzed using a Molecular Dynamics Typhoon PhosphorImager and ImageQuant software. For comparison of RNAP remodeling activity as shown in Figure 5, the data were normalized across multiple gels by determining the total intensity of the remodeling products for any given protein at any given timepoint (see black bar in Figure 5A) and dividing this value by the highest value observed for the wild type activity on each gel (typically the final timepoint). This value therefore represents a relative remodeling activity compared to maximal wild-type activity. The error bars represent the standard error of the mean for wild type (six experiments) or mutant UvrD (four experiments each) respectively.

For transcript release assays, stalled transcription elongation complexes were formed as described above, but using biotinylated template DNA. DNA containing the stalled complexes was bound to streptavidin paramagnetic beads (NEB) by incubating each reaction with beads taken from an equal volume of bead suspension (and washed twice with repair buffer) for 10 min at 20°C. The beads were then washed three times in equal volumes of repair buffer to remove unbound DNA and RNAP. 1 μM UvrD or UvrDΔC was added to 20 μl aliquots of the reaction where indicated and reactions were incubated for 5 min at 37°C. Transcription reactions were chased by adding 100 μM NTPs and 10 μg/ml rifamipicin for 5 min at 37°C. A magnet was used to separate pellet and supernatant fractions and the supernatant was added to 20 μl urea stop mix (fraction S). The pellet was resuspended in 20 μl repair buffer and added to 20 μl urea stop mix (fraction P).

For transcript-release experiments in which backtracking was analyzed with GreB, reaction volumes were scaled up and 1 μM UvrD or its derivatives were added to 100 μl aliquots containing stalled transcription elongation complexes. Transcription reactions were chased as described above. A magnet was used to separate pellet and supernatant fractions and 20 μl of the supernatant was added to 20 μl urea stop mix (fraction S). The beads were resuspended in 80 μl repair buffer and a 20 μl sample was added to 20 μl urea stop mix (fraction P). The remainder of the bead suspension was split into 20 μl aliquots. 1 μM GreB was added where indicated and reactions were incubated for 5 min at 37°C. Then 100 μM NTPs were added where indicated and reactions were incubated for 5 min at 37°C. Reactions were stopped with 20 μl urea stop mix. Samples were heated for 3 min at 90°C and resolved on a 15% polyacrylamide/7 M urea denaturing gel. Gels were analysed using a Molecular Dynamics Typhoon in PhosphorImager mode and ImageQuant software.

### ATPase assays

The ATPase activity of UvrD and its derivatives was measured using an enzyme linked assay in which ATP hydrolysis is coupled to NADH oxidation essentially as reported previously (51). However, ATPase assays were modified in that reactions were carried out at 37°C using 1 nM UvrD, 2 mM ATP and 2 μM ssDNA (47 nt). The ATPase activity of PcrA and its derivatives was measured using the same linked assay, according to the method described in (34).

### TFO displacement (DNA translocase) assays

TFO assays were carried out essentially as described in (49). Assays were carried out on a linear plasmid template, pSRTB2EV, a derivative of pSRTB2 (50), in which an EcoRV site had been introduced downstream of the TFO binding site by site-directed mutagenesis. This template was linearised by EcoRV, creating a blunt end 33 bp downstream of the triplex end. Assays were performed in repair buffer and the TFO containing DNA template was incubated with 1 μM UvrD or its derivatives and 100 μM NTP mix. 0.25 mg/ml Proteinase K and 10 mM CaCl_2_ were added to the GSMB stop buffer and reactions were incubated for 30 min at 20°C before loading onto the gel to eliminate bandshifting by UvrD.

## RESULTS

### Structure of a Tudor-like RNA polymerase interaction domain in PcrA

We have shown previously that the CTD of *G. stearothermophilus* PcrA (PcrA-Ct; residues 653–724) is necessary and sufficient for interaction with RNA polymerase using affinity pulldown assays from extracts of *B. subtilis* (14) (see Supplementary Figure S1). Secondary structure predictions using Jpred (52) suggested that this region of the protein includes a significant region of natively disordered protein that is poorly conserved. However, this is followed by a very highly conserved region that is predicted to consist entirely of beta-sheet (residues 673–724). Furthermore, the domain identification algorithms Ginzu (53) and Phyre2 (54) both predict that this beta-sheet region will fold into a Tudor-like domain. Despite very low primary structure homology, these algorithms identify RapA and CarD respectively as templates for homology modelling. Interestingly, these are both bacterial RNAP interaction partners containing conserved Tudor-like domains (55,56). Therefore, we hypothesized that the extreme C-terminus of PcrA adopts a Tudor fold and that this interacts directly with RNAP. To test this idea, we designed a new shorter version of the PcrA CTD using the homology models as a guide. This construct, which we call PcrA-short Ct (PcrA-sCt), includes residues 673–724 of the native PcrA protein. Moreover, to produce the CTD of PcrA in greater quantities than we had achieved previously using a biotin-tag (14), we engineered plasmids for expression of PcrA-Ct and PcrA-sCt with a cleavable histidine tag at the N-terminus. Using this system, we were able to obtain large amounts of highly pure protein either with or without the tag (Supplementary Figure S2). To confirm that the shorter CTD construct retained the ability to interact with RNAP, we performed affinity pulldown experiments using the his-tagged PcrA-sCt protein as bait and nucleic acid-depleted cell extracts as prey (14). As expected, the original PcrA-Ct construct efficiently pulled down RNAP from a *B. subtilis* extract in a dose-dependent manner. In agreement with our hypothesis, the shorter PcrA-sCt construct retained the ability to pulldown RNAP, and the efficiency was comparable to PcrA-Ct (Supplementary Figure S2). For reasons that will be discussed below, we also investigated whether the PcrA CTD was able to bind to DNA. However, no interaction between the PcrA CTD and either single- or double-stranded DNA was detected using gel shift assays (Supplementary Figure S3).

**Figure 1.**
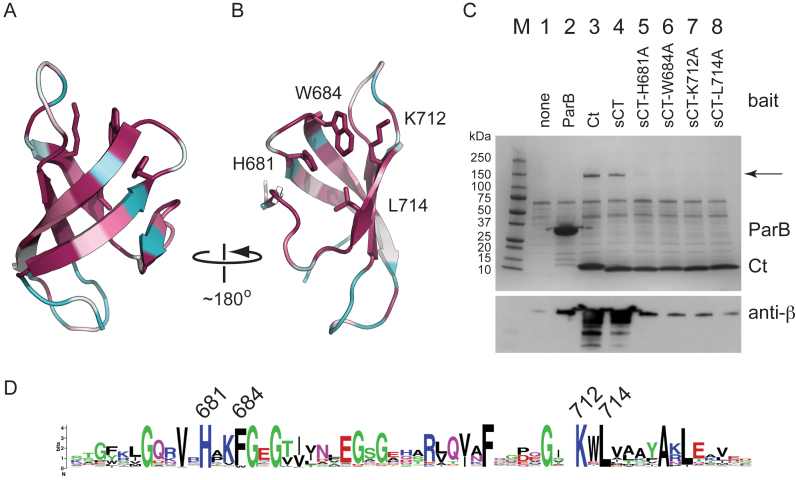
The crystal structure of an RNAP interaction domain in PcrA helicase. (**A**) Crystal structure of the Tudor-like CTD of PcrA. The co-ordinates have been deposited at the PDB under PDB ID code: 5DMA. The structure is colored according to residue conservation with the least conserved residues in cyan, through white, to most conserved residues in magenta. The protein is shown in ribbons format with side chains only shown for the amino acids which were selected for site-directed mutagenesis studies. (**B**) A second view of the structure, facing the putative RNAP binding surface. (**C**) Affinity pulldown assays using the his-tagged proteins indicated as bait and *Bacillus subtilis* cell extract as prey. The upper panel shows an SDS-PAGE gel analysis of the cell extract following pulldown using baited magnetic beads. The bait protein is indicated at the top of the gel and the arrow indicates the position of the β and β΄ subunits of RNAP. The lower panel shows western blot analysis of the same gel using a monoclonal antibody against the β subunit of RNA polymerase. In addition to a mock pulldown, the pulldown with ParB (a centromere binding protein that is not known to interact with RNAP) is included as a second negative control for non-specific pulldown of RNAP. (**D**) Weblogo format multiple sequence alignment of the CTD of ∼250 PcrA/UvrD homologues. The residues that were mutated in this study are highlighted using their residue numbers from the *G. stearothermophilus* PcrA protein.

**Figure 2.**
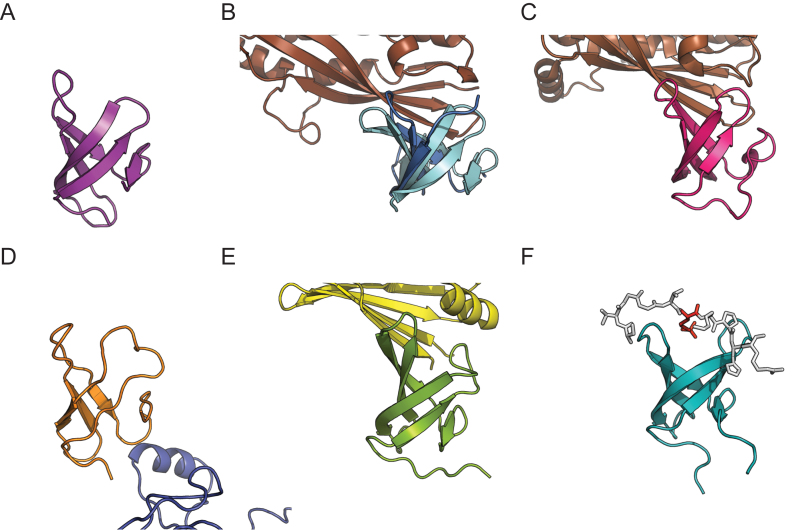
Comparison of Tudor-like domains and their interactions with partner proteins. (**A**) Structure of the CTD of PcrA (PDB: 5DMA; this work). (**B**) Superimposition of the RNAP interaction domain of *E. coli* Mfd (cyan) (PDB: 2EYQ; (57)) with a complex between the Mfd RID (blue) and the β1 domain of RNAP polymerase (brown) (PDB: 3MLQ; (60)). (**C**) The complex of CarD (red) bound to the RNAP β subunit (brown) (PDB: 4KBM; (58)). (**D**) Complex between the second Tudor domain of RapA (orange) and the β΄ subunit of RNAP (blue) (PDB:4S20; (59)). (**E**) Complex between the Tudor-like domain of NusG (green) and ribosomal protein S10 (yellow) (PDB:2KVQ; (63)). (**F**) The PHF1 protein (turquoise) in complex with a peptide (white) shown in stick format and containing a trimethylated lysine residue (red) (PDB:4HCZ; (64)).

Crystals of PcrA-sCt (with the histidine tag removed) were obtained using the sitting drop vapor diffusion method. The structure was solved using a heavy atom derivative at a final resolution of 1.5 Å (*R*_free_ = 22.2%) (Figure 1). The final model includes all of the residues of the native PcrA sequence (W673 to V724) and an N-terminal glycine from the tag linker region. As predicted, the extreme C-terminal region of PcrA adopts a Tudor-like fold consisting of five anti-parallel beta strands that form a twisted β sheet. It closely resembles other bacterial Tudor domains such as those found in RapA, CarD and Mfd (Figure 2) (57–61). A search with DALI (62) also reveals strong similarity to NusG (63) and to eukaryotic ‘histone readers’ including PHF1 (64). These histone readers are responsible for the recognition of methylated lysine residues in chromatin. Many structures of Tudor domains interacting with their partner proteins or peptides have shown that one particular face of the fold is often responsible for the interaction (65), although there are apparent exceptions including RapA (59) (Figure 2). In histone readers, this face includes an ‘aromatic cage’ that typically accepts the methylated side chain of a lysine residue. Interestingly, some key residues that form this aromatic cage appear to be equivalent in PcrA (eg W684, F705). However, PcrA also features a charged lysine residue (K712) in this region. Although not common for Tudor domains in general, this lysine residue is strongly conserved within PcrA/UvrD orthologues (Figure 1D). We were especially interested to compare our structure with the complex of the Mfd Tudor domain bound to RNAP, because PcrA/UvrD has been reported to function in an alternative TCR pathway (1). The structure of *Thermus thermophilus* Mfd bound to RNAP shows that it binds to the so-called β1 region (60) and this is also true of the Tudor domain found in *Mycobacterium tuberculosis* CarD (58,61). In both of those structures, the Tudor domain forms a continuous anti-parallel beta-sheet with the partner protein, which is further stabilized by side-chain interactions. Conserved residues that are important for interaction with RNAP include a lysine residue (K360 in *T. thermophilus* Mfd) that is somewhat similarly positioned to the aforementioned K712 in PcrA (57). However, it should be noted that there is no apparent sequence homology between the Tudor-like domains of PcrA and Mfd.

### Conserved amino acids required for binding RNAP

Several highly conserved residues cluster together on the surface of the PcrA Tudor fold that frequently forms a protein:protein interface in other systems (Figures 1 and 2). We reasoned that these residues might be directly involved in the interaction with RNA polymerase. To test this hypothesis, we individually mutated several of them (H681A, W684A, K712A and L714A) and tested the ability of the resulting CTD constructs to interact with RNAP in pulldown assays. The mutant PcrA-sCt proteins were purified using the same method as for wild type and their CD spectra were all characteristic of β sheet as expected, suggesting normal global folding (Supplementary Figure S4). The pulldown assays revealed that each of the single mutant proteins had a severely reduced ability to bind to RNAP (Figure 1C). Indeed, western blotting of the gels with an anti-RNAP subunit antibody suggested the binding was marginally above a ‘no bait’ control and comparable to a second negative control experiment using *B. subtilis* ParB (a protein not known to bind RNAP).

**Figure 3.**
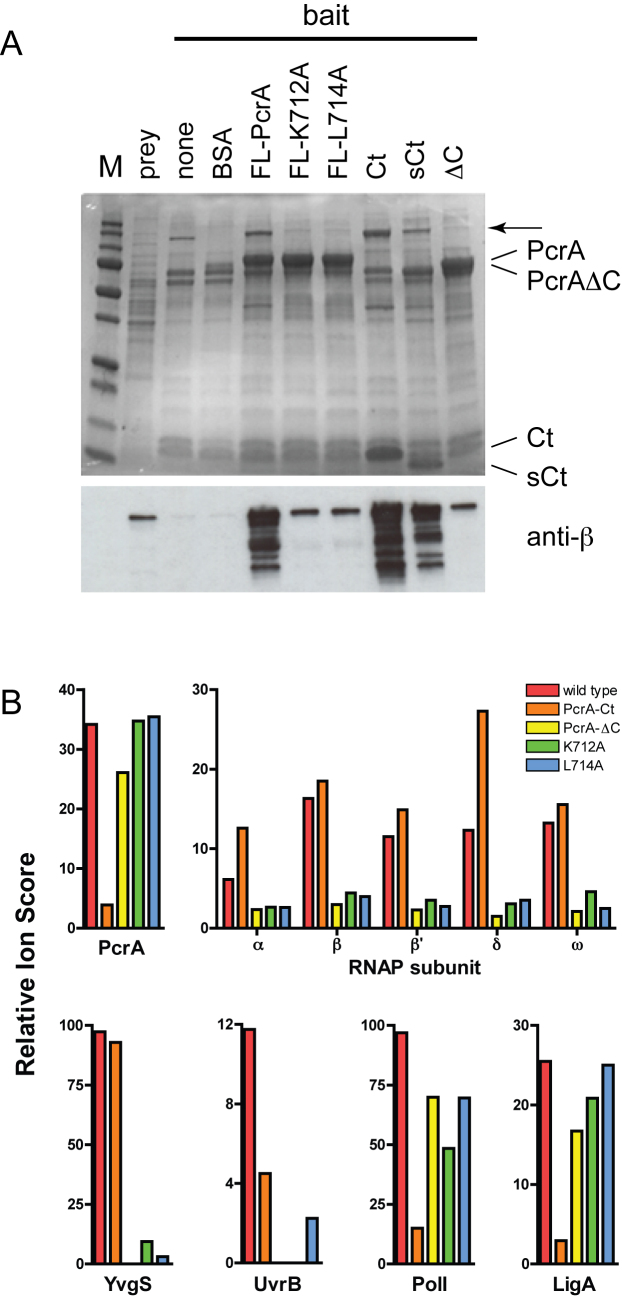
Mutation of the PcrA CTD reduces binding to RNAP. (**A**) Affinity pulldown assays using the biotinylated proteins as bait and *B. subtilis* cell extract as prey. The upper panel shows an SDS-PAGE gel analysis of the cell extract following pulldown using baited magnetic beads. The arrow signifies the position of the β and β΄ subunits of RNAP. The lower panel shows analysis of the same gel using a monoclonal antibody against the β subunit of RNAP. Note that the K712A and L714A mutations were made in the context of full length (FL) PcrA. (**B**) Quantification of the pulldown experiments using mass spectrometry using the bait proteins indicated and a ‘no bait’ control as the reference. The Relative Ion Score value shown is the total ion score for the prey divided by the equivalent value in the control: a measure of relative abundance of the prey. The enrichment of PcrA in the pulldown samples reflects the presence of the bait protein and acts as an internal control. Selected results are shown for the subunits of RNAP and for several other proteins that are discussed in the text. Further and more detailed results are shown in Supplementary Table S1.

The K712A and L714A mutations were also made in the context of a full length biotinylated PcrA construct used in our previous studies. These mutant proteins displayed a greatly reduced ability to interact with RNAP, but the binding was reproducibly higher than background (Figure 3A). Moreover, as has been shown previously (14), the complete deletion of the CTD from the full length protein dramatically reduces binding to RNAP. In order to validate these experiments and to test for protein interactions of the CTD in an unbiased fashion, we also analysed the pulldown experiments using mass spectrometry. Relative quantification of prey proteins was performed by comparing total ion scores using the ‘no bait’ pulldown as a control (Figure 3B and Supplementary Table S1). The relative ion score for the PcrA bait acts as an internal control, showing enrichment over control for full length constructs, and a reduced enrichment for the PcrA-Ct construct as would be expected based on the different polypeptide lengths. The results for the wild type PcrA bait reproduced our previously published experiments showing that it interacts with many proteins in the cell extract (see also (14) and Supplementary Table S1 for full details). Prominently these include both core and accessory subunits of RNA polymerase (α, β, β΄, δ, ω), as well as a variety of sigma factors. Other enriched proteins of interest include YvgS/HelD (a SF1 helicase also known to associate with RNAP), UvrB (a known PcrA binding partner involved in NER), DNA pol I (a DNA repair specific and bypass polymerase) and LigA (an NAD+-dependent DNA ligase originating from the same operon as PcrA) (Figure 3B). In good agreement with the western blotting analysis, mass spectrometry showed that mutation (K712A or L714A) or removal of the PcrA CTD substantially reduced or almost eliminated the interaction with core RNAP subunits respectively. These data show that the mutated residues are critical for the RNAP binding function of the CTD, and that the apparent residual binding we have observed probably occurs at a different site in the PcrA protein. Interestingly, a concomitant reduction in several other interaction partners including UvrB and YvgS was also detected. This is consistent with two possibilities that cannot be distinguished based on these experiments. Either the CTD of PcrA is important for direct interaction with all of these proteins, or the other proteins are associated with the RNAP subunits that are pulled down by PcrA. In this respect it should be noted that PcrA/UvrD interacts directly with RNAP and UvrB (1,14,15), and that YvgS/HelD interacts directly with RNAP (66). In distinct contrast, the removal or mutation of the CTD of PcrA did not affect the apparent interaction with DNA pol I or LigA, whereas the CTD alone bound poorly to these proteins, showing that they must interact mainly with the N-terminal region of PcrA. Importantly, this observation confirms that the mutant PcrA proteins remain largely folded, as would be expected based on CD analysis of the CTD variants alone (see above). This assertion is further supported by analysis of the DNA-dependent ATPase activity, which is similar or slightly better than wild type for both mutant proteins (Table 2).

**Table 2.** ssDNA-dependent ATPase activity of PcrA and UvrD

Protein	ATPase (s^−1^)^a^
Wild type PcrA	12.1 ± 0.8
PcrA K712A	15.8 ± 0.7
PcrA L714A	18.9 ± 1.8
Wild type UvrD	81.1 ± 3.0
UvrDΔC	194.9 ± 7.5
UvrD K708A	82.8± 19.8

^a^ATP hydrolysis was measured using a coupled assay as described in the Materials and Methods under conditions of saturating ATP and ssDNA. The values reported are the mean turnover number and the standard error of the mean for three independent experiments.

### The UvrD CTD is important for the remodeling of RNAP transcripts

Due to differences in the manner in which it was discovered in the model organisms *B. subtilis* and *E. coli*, the helicase studied here is often annotated as PcrA in Gram-positive organisms and UvrD in Gram–negative organisms. It was therefore of considerable interest to us that *E. coli* UvrD was recently shown to induce backtracking of RNAP *in vitro*, causing it to slide backwards on the DNA and bringing about the displacement of the 3΄ end of the RNA from the active site (1). To reproduce this activity and to probe the potential role of the CTD, *in vitro* transcription reactions were performed in which wild type or mutant UvrD was incubated with RNAP (both from *E. coli*) that had been stalled 20 nt downstream of the T7A1 promoter by omission of UTP. The reaction was then ‘chased’ with NTPs, and rifampicin was added to ensure single round conditions.

**Figure 4.**
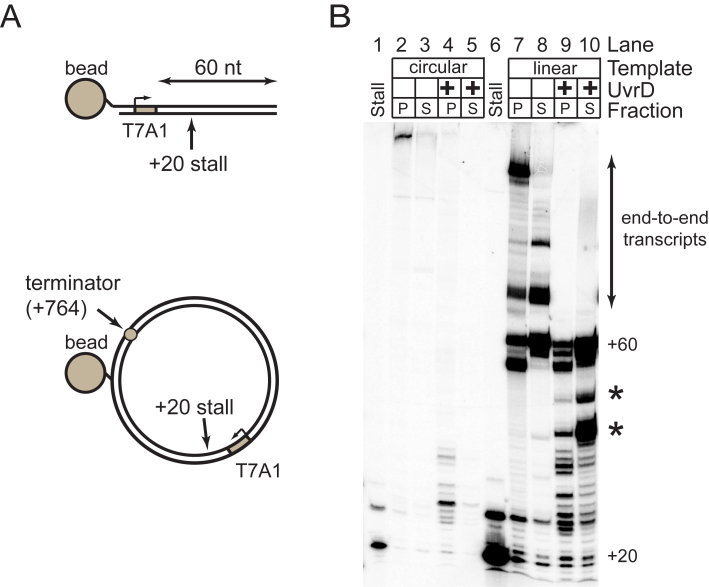
UvrD remodels RNAP transcripts on circular and linear DNA templates. (**A**) Schematic of the linear and circular DNA templates used for transcription reactions in this work. (**B**) Remodeling assay for both circular and linear DNA templates as indicated. Reactions were performed either with or without 1 μM UvrD as described in the Materials and Methods. Biotinylation tags in the template DNA molecules allow the pulldown of the template into a pellet fraction (P) leaving a supernatant fraction (S) which only contains transcripts released into solution. The stall lanes show the stalled transcript product at +20 without the addition of chase nucleotides. All other lanes show transcripts formed following re-initiation of transcription from +20 with a nucleotide chase. Asterisks highlight the position of the principal transcripts that are released into solution by the action of UvrD.

In initial experiments, both circular and linear DNA templates were used, and these were also biotinylated so that displacement of RNAP could be monitored by examining the release of the transcript into the supernatant using a pulldown approach (Figure 4A). In the absence of UvrD, the RNAP forms long transcripts indicative of processive transcription elongation (Figure 4B; lanes 2, 3, 7 and 8). Shorter RNA transcripts are formed in a UvrD-dependent manner regardless of whether the template is circular or linear, and these transcripts generally remain in the pellet fraction (Figure 4B). This is consistent with UvrD causing backtracking of RNAP as reported previously (1), and this was confirmed by adding the transcript cleavage factor GreB (which removes the extruding 3΄ portion of the nascent RNA) and then chasing the reaction with NTPs to generate full length transcripts (Supplementary Figure S5, lanes 1–13, see figure legend for details). In addition to backtracking, we also observed efficient UvrD-dependent displacement of short RNA transcripts into the supernatant, and this phenomenon was only observed with the linear template (Figure 4B, see asterisks). Interestingly, the size of these released transcripts is equivalent to the size of the major GreB-cleavage products (Supplementary Figure S5, compare lanes 9 and 11), which are indicative of favored backtracking positions for RNAP on the template. In the absence of UvrD, several transcripts that are longer than the distance from the promoter to the end of the template were observed. Such transcripts result from transfer of RNAP from an end of one DNA molecule to an end of another (67). Interestingly, addition of UvrD abolishes this end-to-end transfer, possibly by backtracking RNAP away from the DNA end, or by dissociating it from the DNA altogether. Together, these experiments show that UvrD is capable of remodeling transcription elongation complexes *in vitro*, by promoting both the reversible backtracking of RNAP, and the premature release of short RNA transcripts from linear templates.

**Figure 5.**
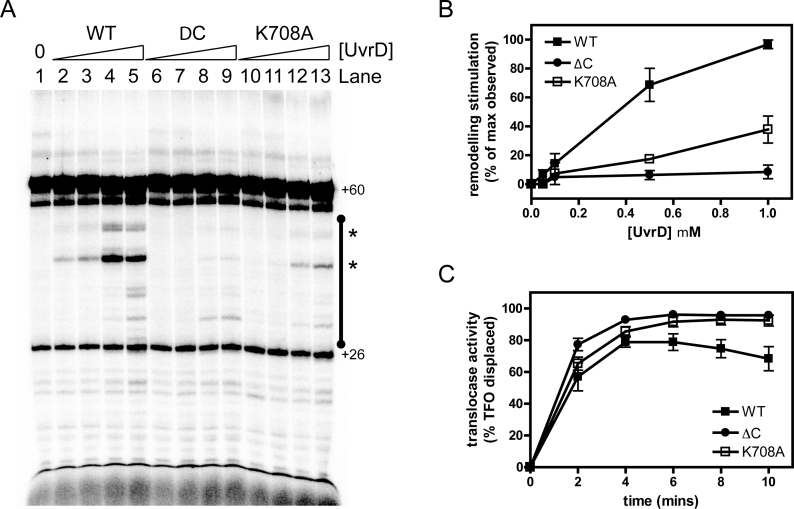
Mutation or removal of the CTD reduces UvrD-dependent remodeling of RNAP transcripts, but not DNA translocase activity. (**A**) Transcript remodeling assay on linear template DNA. An example gel is shown which has been uniformly contrast enhanced. Lane 1 shows the transcripts arising from the activity of RNAP on a linear DNA construct containing a T7A1 promoter (Figure 4A) in the absence of UvrD. The major product is the result of transcription to the end of the template that results in a 60mer which is indicated on the gel. Lanes 2–5 show the effects of adding increasing concentrations of wild type UvrD (0.05, 0.1, 0.5, 1 μM), resulting in many shorter transcripts in an area of interest marked by the black bar. The bands that are marked with an asterisk are those which are released into free solution by the action of UvrD (see Figure 4 for details). (**B**) Quantification of the data shown in panel A for the region of interest shown by the black bar. The data were normalized as described in the methods to provide a measure of the RNAP transcript remodeling activity of the mutant UvrD proteins relative to wild type. The error bars represent the standard error of the mean for six or four independent experiments for wild type or mutant UvrD constructs respectively. (**C**) The DNA translocase activity of wild type and mutant UvrD proteins was measured with a triplex displacement assay as described in the Materials and Methods. The error bars represent the standard deviation about the mean for four or three independent experiments for wild type or mutant UvrD constructs respectively.

We next performed experiments on free linear template DNA using mutant UvrD proteins in which the CTD was either removed or mutated (Figure 5). The very strong conservation of primary sequence in the CTD (Supplementary Figure S1) allows the facile design of mutations in *E. coli* UvrD (UvrDΔC or UvrD^K708A^) that are equivalent to those we have studied in PcrA. In the absence of UvrD, RNAP transcribed the template DNA to produce products which correspond to transcription to the end of the template (Figure 5A; lane 1 +60). As expected, in the presence of wild type UvrD a series of shorter RNA transcripts were observed (Figure 5A, compare lane 1 with lanes 2–5). This effect was UvrD dose-dependent, but less efficient than has been reported previously (1). On these substrates, UvrD-dependent RNAP remodelling was substantially reduced in the absence of the CTD (Figure 5A and B, compare lanes 2–5 with lanes 6–9). However, it was still possible to observe backtracking of RNAP on linear DNA templates using this mutant, especially when bound to streptavidin coated magnetic beads (Supplementary Figure S5). The deletion of the CTD is particularly effective at decreasing the premature release of RNA into solution that is observed on linear templates (Figure 5A and Supplementary Figure S5, see asterisks). Mutation of the highly conserved lysine in the CTD (K708A) also reduced the remodeling of RNAP-RNA complexes relative to wild type, albeit not to the same extent as does the complete removal of the CTD (Figure 5A and B).

Together, these experiments indicate that the CTD is important but not essential for catalysing backtracking and RNA release from RNAP during transcription *in vitro*. To eliminate the possibility that the mutant proteins were simply unfolded, or that the observed remodeling defects reflected a reduced ability of the mutant proteins to move along DNA we assayed for ATPase and DNA translocase activity. These experiments showed that UvrD proteins in which the CTD had been either mutated or deleted retained ssDNA-dependent ATPase and translocase activities that were either comparable to, or even better than, wild type activity (Table 2 and Figure 5C). This is broadly consistent with previous experiments on a UvrD protein with a 40 amino acid C-terminal deletion which displayed wild type ATPase and helicase activity (68).

## DISCUSSION

Superfamily I helicases are highly abundant and often multi-functional enzymes playing diverse roles in bacterial nucleic acid metabolism. Interestingly, several recent studies have shown that these enzymes function at the interface of DNA replication, transcription and repair. In rapidly dividing bacterial cells, the replication and transcription of DNA occur on the same template at the same time. Conflicts between the two systems are inevitable and can lead to genomic instability, and so cells have developed systems that either reduce their occurrence or minimize their impact (reviewed in (69)). For example, some Superfamily I helicases have been shown to help the replisome bypass physical barriers including transcription complexes in various model organisms. However, the mechanisms by which they do so are only recently becoming apparent and may vary widely. In *E. coli*, the SF1 helicases Rep, UvrD and DinG have all been shown to resolve replication:transcription conflicts (26,27,70). Rep functions as a component of the replisome itself, by associating with the replicative helicase DnaB, and loss of this interaction results in transcription:replication conflicts (26,71). In distinct contrast, UvrD interacts directly with RNAP (1), and an equivalent interaction has also been demonstrated in the orthologous PcrA enzyme (14,21,72). However, recent work in *B. subtilis* shows that deletion of the PcrA CTD does not affect its essential role in resolving replication:transcription conflicts, whereas elimination of its ATPase/helicase activity does (27). Although perhaps surprising, this result is consistent with the long standing observation that Rep and UvrD share an essential function in *E. coli* (73,74), despite the fact that Rep does not interact with RNAP. Nevertheless, the extremely high conservation of the PcrA/UvrD CTD region would suggest either that there is some unappreciated complexity in observing a phenotype associated with its deletion and/or that it has a different role that is also (presumably) related to transcription. Indeed, recent experiments have suggested that UvrD can backtrack RNAP *in vitro* and *in vivo* (1,2). We have shown here that this backtracking function is perturbed, albeit not entirely eliminated, by deletion or mutation of the CTD. Therefore, this work potentially identifies separation of function mutants that can be used to study the backtracking role of PcrA/UvrD specifically (25). In this respect, it is interesting and important to note that the loss of the PcrA/UvrD CTD has no apparent effect on nucleotide excision repair, mismatch repair or the resolution of transcription:replication conflicts *in vivo* (see Supplementary Figure S6 and accompanying legend) (15,27,68,75). This implies that efficient RNAP remodeling is not important for any of these processes, at least in certain circumstances. A key challenge for future experiments will be to rationalise how the extreme multi-functionality displayed by helicases like PcrA/UvrD relates to the spectrum of protein:protein interactions that they can form, and how these different aspects of their function are regulated.

We have further defined the minimal RNAP polymerase interaction domain in the C-terminal region using *G. stearothermophilus* PcrA as a model system. We have shown for the first time that this adopts a Tudor fold in common with several other bacterial proteins involved in modulating transcription. Removal of the CTD of PcrA causes either no reduction or a modest reduction in ATPase, helicase and translocase activity *in vitro* (14,32). A similar analysis of UvrD showed no change in translocase activity and either no change or a moderate increase in ATPase (this work and (15,68,75)) Moreover, our biochemical analysis shows that the isolated CTD of PcrA does not bind to either single- or double-stranded DNA (Supplementary Figure S3). Therefore, despite the location of this domain in the vicinity of the displaced strand during DNA unwinding, it does not seem to be involved in directly stimulating helicase activity, and may even have an attenuating effect on the ATPase. Simple point mutations in the CTD of PcrA greatly reduce RNAP binding but have no significant effect on the ssDNA-dependent ATPase activity. These mutations map to a well-conserved surface on the CTD of PcrA/UvrD orthologues. It is noteworthy that the same region of the Tudor fold is frequently observed to be the interaction interface for many other Tudor domains that are quite diverse at the level of primary structure. Given that Tudor domains are well-characterized as readers of methylated lysines in eukaryotic chromatin, a highly speculative possibility is that PcrA/UvrD might interact with RNAP following post-translational modifications of lysines or arginines. These are abundant in bacterial proteins including RNAP but their function is usually unclear (76,77).

We do not know precisely where the CTD of PcrA/UvrD engages RNAP. Two-hybrid data and far western blots have suggested that the PcrA CTD contacts the N-terminal region (aa 1–400) of the β subunit (29). Given that related Tudor domains in Mfd and CarD/CdnL family proteins make contact with the β1 region (which is found within aa 1–400), an intriguing possibility is that PcrA/UvrD and Mfd compete for the same interaction patch on RNAP. However, we do not currently favor this possibility because we do not observe a dominant negative effect of the *E. coli* UvrD CTD upon Mfd function *in vitro* (data not shown). It is also quite possible that the C-terminal Tudor domain is not the only determinant of the RNAP interaction, and feasible that the details of the interaction differ between the UvrD and PcrA proteins studied here. Such factors would complicate the interpretation of any phenotypic analysis, and might also explain why the PcrA/UvrD mutants used in this study retain a limited ability to bind or backtrack RNA polymerase. Indeed, far western blots have suggested that an additional region in the N-terminus of PcrA is important for an interaction with the β΄ subunit of RNAP in *B. subtilis* (29). Moreover, crosslinking experiments with *E. coli* UvrD and RNAP also suggest an interface involving the N-terminal helicase region (1). Further support for a more extensive interface is provided by analogy with RapA. A structure of RapA bound to RNAP shows that, although its Tudor domains are important for the inter-protein interactions as expected, the entire RapA protein including the core helicase regions also plays a role in the interactions (59). A final complexity is that the interaction could be modulated by a regulatory signal. For example, the ability of UvrD to backtrack RNAP has recently been shown to be enhanced by the presence of the small molecule alarmone ppGpp: this effect is likely due in part to changes in the properties of RNAP, but may also reflect an altered interaction between UvrD and RNAP (2). These questions will be the subject of further study.

## Supplementary Material

Supplementary Data
