# Supplementary material for: The structure and function of an RNA polymerase interaction domain in the PcrA/UvrD helicase
Source: Nucleic Acids Res. 2017 Feb 4;45(7):3875–87. doi: 10.1093/nar/gkx074 (PMC5397179; doi:10.1093/nar/gkx074)

The structure and function of an RNA polymerase interaction domain in the PcrA/UvrD helicase

SUPPLEMENTARY INFORMATION

Sanders, K.*[1], Lin, C-L.*[2], Smith, A.J.*[1], Cronin, N.[2], Fisher, G.[1], Eftychidis., V.[3], McGlynn, P.[3], Savery, N.J.[1], Wigley, D.B.[2] and Dillingham, M.S.[1#]

[1]DNA:Protein Interactions Unit, School of Biochemistry, Biomedical Sciences Building, University of Bristol, BS8 1TD, UK. [2]Institute of Cancer Research, Chester Beatty Laboratories, 237 Fulham Road, London SW3 6JB, UK and Section of Structural Biology, Department of Medicine, Imperial College London, South Kensington Campus, London SW7 2AZ, UK. [3]Department of Biology, University of York, Wentworth Way, York YO10 5DD, United Kingdom.

SUPPLEMENTARY METHODS

Electrophoretic Mobility Shift Assays

The DNA-binding activity of purified PcrA-sCt was analysed by TBE-PAGE gel shift. Serial dilutions of PcrA-sCt, to the indicated concentrations, were incubated with 20 nM radiolabelled substrate (147 base ssDNA oligonucleotide or 147 base pair dsDNA as indicated) in a buffer containing 50 nM HEPES-KOH pH 7.5, 100 mM KCl, 2.5 mM $MgCl_2$, 0.1 mg/mL BSA, 1 mM DTT and 2.5 % (v/v) Ficoll in a 20 µl reaction volume. Samples were incubated at room temperature for 30 mins followed by 5 mins on ice. 10 µl of each were loaded onto a 6% acrylamide/bis-acrylamide (29:1) gel in 90 mM Tris, 150 mM Boric acid (final pH 7.5), 1 mM EDTA. Gels were pre-run at 150 V, 4°C for 30 mins in a buffer identical to their composition, and run post-loading at 150 V, 4°C for 1 hr. For imaging, gels were dried under vacuum and exposed to a phosphor screen, which was subsequently scanned by a Phosphor-Imager (Typhoon FLA 9500, GE Healthcare Life Sciences). ParB protein was used as a positive control for DNA binding.

Circular dichroism spectroscopy

CD spectroscopy was used to assess the folding of wild type and mutant PcrA CTDs. CD spectra were collected at 20°C using a 0.1 cm quartz cuvette in a JASCO J-810 spectropolarimeter. Samples were prepared by dialysing untagged proteins into phosphate buffered saline (PBS, 8.2 mM disodium hydrogen phosphate, 1.8 mM potassium dihydrogen phosphate, 137 mM sodium chloride and 2.7 mM potassium chloride (pH 7.4)) at the concentrations indicated. Data was acquired across a 190-260 nm absorbance scan using a band width of 1.00 nm, time constant of 1

s, scan rate of 100 nm/min, and accumulation of 64 scans, and then normalised to molar ellipticity (MRE ($deg.cm^2.dmol^{-1}$)) by calculation of the concentration of peptide bonds and the cell path length. A buffer only baseline was subtracted from all datasets.

Mass spectrometry sample preparation

Each gel lane was cut into slices and each slice subjected to in-gel tryptic digestion using a ProGest automated digestion unit (Digilab UK). The resulting peptides were fractionated using a Dionex Ultimate 3000 nanoHPLC system in line with an LTQ-Orbitrap Velos mass spectrometer (Thermo Scientific). In brief, peptides in 1% (vol/vol) formic acid were injected onto an Acclaim PepMap C18 nano-trap column (Dionex). After washing with 0.5% (vol/vol) acetonitrile 0.1% (vol/vol) formic acid peptides were resolved on a 250 mm × 75 μm Acclaim PepMap C18 reverse phase analytical column (Dionex) over a 150 min organic gradient, using 7 gradient segments (1-6% solvent B over 1minute, 6-15% B over 58 minutes, 15-32% B over 58 minutes, 32-40% B over 3minutes, 40-90% B over 1minutes, held at 90% B for 6 minutes and then reduced to 1%B over 1 minute.) with a flow rate of 300 nl min^{-1}. Solvent A was 0.1% formic acid and Solvent B was aqueous 80% acetonitrile in 0.1% formic acid. Peptides were ionized by nano-electrospray ionization at 2.1 kV using a stainless steel emitter with an internal diameter of 30 μm (Thermo Scientific) and a capillary temperature of 250°C. Tandem mass spectra were acquired using an LTQ- Orbitrap Velos mass spectrometer controlled by Xcalibur 2.1 software (Thermo Scientific) and operated in data-dependent acquisition mode. The Orbitrap was set to analyze the survey scans at 60,000 resolution (at m/z 400) in the mass range m/z 300 to 2000 and the top twenty multiply charged ions in each duty cycle selected for MS/MS in the LTQ linear ion trap. Charge state filtering, where unassigned precursor ions were not selected

for fragmentation, and dynamic exclusion (repeat count, 1; repeat duration, 30s; exclusion list size, 500) were used. Fragmentation conditions in the LTQ were as follows: normalized collision energy, 40%; activation q, 0.25; activation time 10ms; and minimum ion selection intensity, 500 counts. The raw data files were processed and quantified using Proteome Discoverer software v1.2 (Thermo Scientific) and searched against the UniProt *B.subtilis* database using the SEQUEST (Ver. 28 Rev. 13) algorithm. Peptide precursor mass tolerance was set at 10 ppm, and MS/MS tolerance was set at 0.8 Da. Search criteria included carbamidomethylation of cysteine (+57.0214) as a fixed modification and oxidation of methionine (+15.9949) as a variable modification. Searches were performed with full tryptic digestion and a maximum of 1 missed cleavage was allowed. The reverse database search option was enabled and all peptide data was filtered to satisfy false discovery rate (FDR) of 5%. The Proteome Discoverer software generates a reverse "decoy" database from the same protein database and any peptides passing the initial filtering parameters that were derived from this decoy database are defined as false positive identifications. The minimum cross-correlation factor (Xcorr) filter was readjusted for each individual charge state separately to optimally meet the predetermined target FDR of 5% based on the number of random false positive matches from the reverse decoy database. Thus each data set has its own passing parameters.

Phenotypic analysis of UvrDΔC (UvrD$^{1-647}$)

Sensitivity to UV light was assayed as described (1). The frequency of rifampicin resistant colonies was monitored by inoculating 10 ml of LB with 0.1 ml of an overnight LB culture of the relevant strain and growing with shaking at 37°C to an A650 of 0.4. 10-fold serial dilutions of the culture were made using 56/2 salts and 5 μl of each deletion spotted onto LB plates to

estimate total numbers of colony-forming units/ml after growth overnight at 37°C. Estimation of the numbers of rifampicin resistant colonies was made by plating 100 µl of the mid-log phase culture onto LB containing 15 µg/ml rifampicin. Additionally 1 ml and 5 ml of the same culture were pelleted by centrifugation and each pellet resuspended in 100 µl of 56/2 salts prior to plating out onto LB containing 15 µg/ml rifampicin. Numbers of rifampicin resistant colonies were estimated after overnight growth at 37°C. The fraction of rifampicin resistant colonies was calculated by dividing the number of rifampicin resistant colonies by the total number of colonies after correcting for dilution factors. Viability of *uvrd*/*rep* cells was monitored using a plasmid loss assay as described previously (2). The strains used were: (i) JA031 (pAM403 *(lac*$^{+}$ *rep*$^{+}$*)* / *ΔlacIZYA uvrD*$^{+}$ *Δrep*::*cat*); (ii) JA033 (pAM403 *(lac*$^{+}$ *rep*$^{+}$*)* / *ΔlacIZYA ΔuvrD::dhfr Δrep*::*cat*); (iii) VE016 (pAM403 *(lac*$^{+}$ *uvrD*$^{+}$*)* / *ΔlacIZYA uvrD*$^{1\text{-}647}$::<*kan*> *Δrep*::*cat*).

SUPPLEMENTARY FIGURE LEGENDS

Supplementary Table 1- Mass spectrometry analysis of pulldown experiments using PcrA, PcrA CTD, PcrA ΔCTD, PcrA^{K712A} and PcrA^{L714A} as baits.

Total ion scores are used as a measure of abundance when comparing the same prey protein between datasets. The relative ion score (RIS), which is a measure of relative abundance versus the "no bait" control experiment, is shown for each PcrA bait construct. The RIS columns include a red histogram to highlight the high relative abundance scores. Note that, to aid visualisation of the data, an arbitrary high score of 100 has been used in place of a value of infinity in cases where the prey was not detected in the control. Prey proteins that are discussed in the text are highlighted in yellow. The PcrA detection row (which acts as an internal control because it is detecting the bait protein) is highlighted in green. The table shown here contains data in which the wild type RIS value is >2 and the wild type ion score is >10 in order of descending wild type total ion score. The complete raw datasets for these experiments, including values for the whole proteome and a more detailed account of all the mass spectrometry parameters, are available from the corresponding author upon request.

SFigure 1. Structure of the PcrA/UvrD helicase

(A) Crystal structure of PcrA helicase (3PJR; (3)) colour coded by subdomains according to the key. Note that the CTD (purple) is not observed as it is disordered. (B) Primary structure diagram of PcrA indicating the domain organisation. Numbers indicate the positions of domain boundaries. (C) Sequence alignment of *E. coli* UvrD, *B. subtilis* PcrA and *G. stearothermophilus* PcrA. The alignment is colour-coded according to conservation with darker blue segments more

conserved. Note the high conservation of the final ~50 amino acids that fold into a Tudor-like RNAP interaction domain.

SFigure 2. Purification and RNAP binding ability of the PcrA-sCt protein.

(A) Schematic showing the primary structure of wild type PcrA, his-tagged PcrA-Ct and his-tagged PcrA-sCt proteins. The sequence of the tag is shown and the site of cleavage by HRV 3C protease is marked with an asterisk. (B) SDS-PAGE gel showing purified PcrA-sCt protein with the his-tag either intact or cleaved as indicated. (C) Dose-dependent pulldown of RNAP from *B. subtilis* lysate by the his-tagged PcrA-sCt protein is comparable to that by the his-tagged PcrA-Ct protein, which is equivalent to the construct used in our previous studies (4). The position of the β and β' subunits of RNAP is indicated with an arrow.

SFigure 3. Purified PcrA-sCt protein does not bind DNA.

Electrophoretic mobility shift assays were performed as described in the methods using *B. subtilis* ParB protein as a positive control. No DNA binding activity was detected for either single- or double-stranded DNA substrates under these conditions at concentrations up to 5 µM PcrA-sCt.

SFigure 4. The CD spectra for wild type and mutant PcrA sCt domains are similar.

Top panel: CD spectra for the proteins indicated were obtained at 0.25 mg/ml as described in the Materials and Methods. The K712A mutant was not available at this concentration. Lower panel: CD spectra for the proteins indicated were obtained at 0.16 mg/ml. All of the spectra are

characteristic of β sheet as expected based on the crystal structure, and suggest that the mutant proteins are globally folded.

SFigure 5. The UvrD-dependent short RNA transcripts that remain associated with the template DNA are backtracked.

Remodelling assays were performed with either wild type UvrD or UvrDΔC, and using GreB to test for backtracking, as described in the main methods section. Lane 1 shows the stalled transcript product (+20) without addition of the chase nucleotides, whereas lanes 2, 3 and 4 show the transcripts formed following re-initiation of transcription using the chase and pulldown with streptavidin beads. Lane 2 shows the total transcript population (T), Lane 3 shows the free RNA transcripts released into the supernatant (S) and lane 4 shows the transcripts that remain associated with RNAP and the DNA template in the pellet (P). Lane 5 shows the effect of treating the pellet fraction with GreB which cleaves any transcripts that are in a backtracked RNAP complex. Lane 6 shows the effect of a 2nd nucleotide chase which will restart transcription following transcript cleavage by GreB. Lane 7 is a control to show the effect of the 2nd chase step but without addition of the GreB factor. Lanes 8-19 show equivalent experiments performed in the presence of wild type UvrD or UvrDΔC. Asterisks highlight the position of the principal transcripts that are released into solution by the action of UvrD. Note that these correspond with the position of prominent cleavage products formed by the action of GreB.

SFigure 6. Deletion of the C-terminal domain of UvrD does not impact on nucleotide excision repair, mismatch repair or the ability of cells to survive in the absence of Rep.

(A) Δ*uvrD* cells display increased sensitivity to 254 nm UV light as compared with *uvrD*$^{+}$ cells due to a defect in nucleotide excision repair (5) (compare also i with ii). In contrast, a chromosomal allele encoding UvrD lacking the C-terminal 73 amino acids, *uvrD*$^{1\text{-}647}$, does not confer increased sensitivity to UV (compare iii with i). This supports the conclusion that UvrD lacking the C-terminus can function in nucleotide excision repair (6) at least up to the highest UV dose tested here. *uvrD*$^{+}$, *uvrD*$^{1\text{-}647}$ and Δ*uvrD* strains are TB28 (MG1655 *ΔlacIZYA uvrD*$^{+}$)(7), N6632 (MG1655 *ΔlacIZYA ΔuvrD::dhfr*)(2) and VE10 (MG1655 *ΔlacIZYA uvrD*$^{1\text{-}647}$*::<kan>*). (B) Increased spontaneous acquisition of resistance to rifampicin is conferred by a defect in mismatch repair ability, evinced by the elevated frequency of rifampicin resistant colonies formed by Δ*uvrD* cells as compared with *uvrD*$^{+}$ (8) (compare also i with ii). The frequency of rifampicin resistance in *uvrD*$^{1\text{-}647}$ cells is similar to that of *uvrD*$^{+}$ (compare iii with i), indicating that loss of the UvrD C-terminus does not result in a defect in mismatch repair. The strains used are identical to those in A above. (C) Δ*uvrD* Δ*rep* cells are inviable on rich medium since either Rep or UvrD is needed to act as an accessory replicative helicase to aid fork movement along protein-bound DNA (2,9). Viability can be monitored using a very low copy and highly unstable plasmid, pRC7, that encodes the *lac* operon and either *uvrD* or *rep* (2). Retention or loss of pRC7*rep* can be monitored in strains bearing a chromosomal deletion of the *lac* operon by blue/white screening on LB plates containing X-gal and IPTG (2,7). The instability of pRC7 results in a very high rate of plasmid loss in the absence of antibiotic selection for the plasmid but only if the plasmid-less genotype of the strain confers viability, as for *uvrD*$^{+}$ Δ*rep* cells (2) (see also i). Consequently, Δ*uvrD* Δ*rep* cells cannot lose pRC7*rep* (compare ii with i). In contrast, *uvrD*$^{1\text{-}647}$ Δ*rep* cells can form white plasmidless colonies (iii),

indicating that UvrD lacking the C-terminal domain can compensate for the absence of Rep and confer viability. The strains used are shown in the methods.

References

1. Lloyd, R.G. and Buckman, C. (1991) Genetic analysis of the *recG* locus of *Escherichia coli* K-12 and of its role in recombination and DNA repair. *J. Bacteriol.*, **173**, 1004-1011.
2. Guy, C.P., Atkinson, J., Gupta, M.K., Mahdi, A.A., Gwynn, E.J., Rudolph, C.J., Moon, P.B., van Knippenberg, I.C., Cadman, C.J., Dillingham, M.S. *et al.* (2009) Rep Provides a Second Motor at the Replisome to Promote Duplication of Protein-Bound DNA. *Mol. Cell*, **36**, 654-666.
3. Velankar, S.S., Soultanas, P., Dillingham, M.S., Subramanya, H.S. and Wigley, D.B. (1999) Crystal structures of complexes of PcrA DNA helicase with a DNA substrate indicate an inchworm mechanism. *Cell*, **97**, 75-84.
4. Gwynn, E.J., Smith, A.J., Guy, C.P., Savery, N.J., McGlynn, P. and Dillingham, M.S. (2013) The conserved C-terminus of the PcrA/UvrD helicase interacts directly with RNA polymerase. *PLoS One*, **8**, e78141.
5. Ogawa, H., Shimada, K. and Tomizawa, J. (1968) Studies on radiation-sensitive mutants of *E. coli*. I. Mutants defective in the repair synthesis. *Mol. Gen. Genet.*, **101**, 227-244.
6. Manelyte, L., Guy, C.P., Smith, R.M., Dillingham, M.S., McGlynn, P. and Savery, N.J. (2009) The unstructured C-terminal extension of UvrD interacts with UvrB, but is dispensable for nucleotide excision repair. *DNA Repair (Amst)*, **8**, 1300-1310.
7. Bernhardt, T.G. and de Boer, P.A. (2004) Screening for synthetic lethal mutants in *Escherichia coli* and identification of EnvC (YibP) as a periplasmic septal ring factor with murein hydrolase activity. *Mol. Microbiol.*, **52**, 1255-1269.
8. Viswanathan, M., Burdett, V., Baitinger, C., Modrich, P. and Lovett, S.T. (2001) Redundant exonuclease involvement in Escherichia coli methyl-directed mismatch repair. *J. Biol. Chem.*, **276**, 31053-31058.
9. Boubakri, H., de Septenville, A.L., Viguera, E. and Michel, B. (2010) The helicases DinG, Rep and UvrD cooperate to promote replication across transcription units *in vivo*. *EMBO J.*, **29**.

Sanders et al., Supplementary Table 1

Accession	Description	Control Score	WT Score	WT RIS	CTD Score	CTD RIS	deltaC Score	deltaC RIS	K712A Score	K712A RIS	L714A Score	L714A RIS
P37871	DNA-directed RNA polymerase subunit beta' OS=Bacillus subtilis (strain 168) GN=rpoC PE=1 SV=4 - [RPOC_BACSU]	305.75	3518.07	11.51	4555.28	14.90	703.91	2.30	1081.84	3.54	839.20	2.74
P37870	DNA-directed RNA polymerase subunit beta OS=Bacillus subtilis (strain 168) GN=rpoB PE=1 SV=2 - [RPOB_BACSU]	180.09	2938.50	16.32	3329.66	18.49	537.17	2.98	798.47	4.43	715.37	3.97
O34580	ATP-dependent DNA helicase PcrA OS=Bacillus subtilis (strain 168) GN=pcrA PE=1 SV=1 - [PCRA_BACSU]	39.83	1361.33	34.18	155.88	3.91	1039.72	26.11	1383.22	34.73	1411.15	35.43
P20429	DNA-directed RNA polymerase subunit alpha OS=Bacillus subtilis (strain 168) GN=rpoA PE=1 SV=1 - [RPOA_BACSU]	93.01	569.90	6.13	1170.25	12.58	217.57	2.34	244.99	2.63	243.58	2.62
O32215	Helicase IV OS=Bacillus subtilis (strain 168) GN=helD PE=1 SV=1 - [HELD_BACSU]	3.40	331.03	97.47	315.60	92.93	0.00	0.00	32.44	9.55	10.50	3.09
O34996	DNA polymerase I OS=Bacillus subtilis (strain 168) GN=polA PE=3 SV=1 - [DPO1_BACSU]	3.36	325.52	96.92	50.34	14.99	234.81	69.91	162.82	48.48	233.80	69.61
P05653	DNA gyrase subunit A OS=Bacillus subtilis (strain 168) GN=gyrA PE=1 SV=1 - [GYRA_BACSU]	52.41	325.34	6.21	53.50	1.02	301.23	5.75	218.37	4.17	301.72	5.76
P17820	Chaperone protein DnaK OS=Bacillus subtilis (strain 168) GN=dnaK PE=1 SV=3 - [DNAK_BACSU]	133.04	274.88	2.07	102.79	0.77	226.32	1.70	118.31	0.89	157.68	1.19
O06975	Putative sporulation transcription regulator WhiA OS=Bacillus subtilis (strain 168) GN=whiA PE=3 SV=1 - [WHIA_BACSU]	109.29	225.02	2.06	184.84	1.69	154.66	1.42	252.83	2.31	240.40	2.20
Q45598	Uncharacterized protein YydD OS=Bacillus subtilis (strain 168) GN=yydD PE=4 SV=1 - [YYDD_BACSU]	50.59	185.91	3.67	159.06	3.14	156.12	3.09	294.60	5.82	305.46	6.04
P39138	Arginase OS=Bacillus subtilis (strain 168) GN=rocF PE=1 SV=1 - [ARGI_BACSU]	74.85	181.57	2.43	85.68	1.14	176.12	2.35	77.42	1.03	113.11	1.51
O32210	Glyoxal reductase OS=Bacillus subtilis (strain 168) GN=yvgN PE=1 SV=1 - [GR_BACSU]	67.29	171.84	2.55	56.62	0.84	133.36	1.98	40.90	0.61	83.72	1.24
O07906	Uncharacterized HTH-type transcriptional regulator YraN OS=Bacillus subtilis (strain 168) GN=yraN PE=3 SV=1 - [YRAN_BACSU]	45.63	171.39	3.76	120.55	2.64	111.68	2.45	152.02	3.33	181.29	3.97
P37551	Pur operon repressor OS=Bacillus subtilis (strain 168) GN=purR PE=1 SV=1 - [PURR_BACSU]	5.52	157.76	28.57	73.85	13.38	85.52	15.49	139.19	25.21	147.60	26.73
P80865	Succinyl-CoA ligase [ADP-forming] subunit alpha OS=Bacillus subtilis (strain 168) GN=sucD PE=1 SV=3 - [SUCD_BACSU]	66.73	142.32	2.13	64.29	0.96	157.77	2.36	72.17	1.08	95.64	1.43
O31498	DNA ligase OS=Bacillus subtilis (strain 168) GN=ligA PE=3 SV=1 - [DNLJ_BACSU]	5.33	135.61	25.47	15.64	2.94	89.04	16.72	110.99	20.84	133.16	25.01
P07860	RNA polymerase sigma-F factor OS=Bacillus subtilis (strain 168) GN=sigF PE=1 SV=1 - [RPSF_BACSU]	3.26	117.89	36.17	108.44	33.27	43.09	13.22	41.74	12.80	75.05	23.02
P29072	Chemotaxis protein CheA OS=Bacillus subtilis (strain 168) GN=cheA PE=1 SV=2 - [CHEA_BACSU]	43.20	108.02	2.50	77.80	1.80	75.01	1.74	49.29	1.14	53.91	1.25
O06728	Putative phytoene/squalene synthase YisP OS=Bacillus subtilis (strain 168) GN=yisP PE=1 SV=2 - [YISP_BACSU]	0.00	106.33	100.00	73.85	100.00	34.41	100.00	53.33	100.00	62.18	100.00
Q45595	Putative peptide biosynthesis protein YydG OS=Bacillus subtilis (strain 168) GN=yydG PE=4 SV=1 - [YYDG_BACSU]	51.63	104.44	2.02	226.83	4.39	124.25	2.41	218.82	4.24	176.90	3.43
O34863	UvrABC system protein A OS=Bacillus subtilis (strain 168) GN=uvrA PE=3 SV=1 - [UVRA_BACSU]	16.89	104.15	6.17	57.19	3.39	92.36	5.47	126.33	7.48	169.74	10.05
Q45600	Uncharacterized metallophosphoesterase-like protein YydB OS=Bacillus subtilis (strain 168) GN=yydB PE=3 SV=1 - [YYDB_BACSU]	37.61	102.73	2.73	96.67	2.57	84.99	2.26	165.58	4.40	152.53	4.06
O35011	DNA-directed RNA polymerase subunit omega OS=Bacillus subtilis (strain 168) GN=rpoZ PE=3 SV=1 - [RPOZ_BACSU]	7.41	98.01	13.23	115.19	15.55	15.66	2.11	34.00	4.59	18.44	2.49
P35165	RNA polymerase sigma factor SigX OS=Bacillus subtilis (strain 168) GN=sigX PE=1 SV=2 - [SIGX_BACSU]	2.48	94.69	38.17	55.38	22.32	15.15	6.11	43.35	17.47	53.26	21.47
P94541	Ribonuclease HIII OS=Bacillus subtilis (strain 168) GN=rnhC PE=1 SV=2 - [RNH3_BACSU]	43.94	93.02	2.12	122.49	2.79	73.91	1.68	182.51	4.15	147.25	3.35
P39788	Endonuclease III OS=Bacillus subtilis (strain 168) GN=nth PE=3 SV=1 - [END3_BACSU]	20.78	90.04	4.33	58.62	2.82	87.71	4.22	126.88	6.11	158.72	7.64
O34885	Type-2 restriction enzyme BsuMI component YdiS OS=Bacillus subtilis (strain 168) GN=ydiS PE=2 SV=1 - [YDIS_BACSU]	1.68	88.90	53.02	20.66	12.32	141.56	84.43	180.04	107.38	142.50	84.99
P46337	HTH-type transcriptional regulator IolR OS=Bacillus subtilis (strain 168) GN=iolR PE=3 SV=1 - [IOLR_BACSU]	0.00	88.06	100.00	20.39	100.00	84.25	100.00	113.77	100.00	126.47	100.00
O05389	Uncharacterized oxidoreductase YrbE OS=Bacillus subtilis (strain 168) GN=yrbE PE=3 SV=2 - [YRBE_BACSU]	10.88	87.09	8.00	66.16	6.08	153.52	14.11	91.21	8.38	132.50	12.18
O34705	Phospholipase YtpA OS=Bacillus subtilis (strain 168) GN=ytpA PE=1 SV=1 - [PLBAC_BACSU]	33.06	85.59	2.59	129.29	3.91	66.44	2.01	137.50	4.16	105.78	3.20
P12464	DNA-directed RNA polymerase subunit delta OS=Bacillus subtilis (strain 168) GN=rpoE PE=1 SV=1 - [RPOE_BACSU]	6.86	84.58	12.32	187.43	27.30	10.32	1.50	20.99	3.06	24.20	3.53
P08164	NH(3)-dependent NAD(+) synthetase OS=Bacillus subtilis (strain 168) GN=nadE PE=1 SV=5 - [NADE_BACSU]	34.08	83.36	2.45	21.06	0.62	76.76	2.25	20.97	0.62	47.42	1.39
O31656	Uncharacterized protein YkrK OS=Bacillus subtilis (strain 168) GN=ykrK PE=4 SV=1 - [YKRK_BACSU]	17.63	79.52	4.51	44.14	2.50	51.86	2.94	86.84	4.93	72.51	4.11
P50849	Polyribonucleotide nucleotidyltransferase OS=Bacillus subtilis (strain 168) GN=pnp PE=1 SV=3 - [PNP_BACSU]	29.91	74.99	2.51	24.83	0.83	73.13	2.45	13.87	0.46	20.06	0.67
P12042	Phosphoribosylformylglycinamidine synthase subunit PurL OS=Bacillus subtilis (strain 168) GN=purL PE=1 SV=2 - [PURL_BACSU]	34.63	69.36	2.00	41.75	1.21	54.81	1.58	46.61	1.35	94.16	2.72
P23478	ATP-dependent helicase/nuclease subunit A OS=Bacillus subtilis (strain 168) GN=addA PE=1 SV=2 - [ADDA_BACSU]	0.00	69.21	100.00	39.06	100.00	34.72	100.00	48.97	100.00	74.53	100.00
P17869	RNA polymerase sigma-H factor OS=Bacillus subtilis (strain 168) GN=sigH PE=1 SV=1 - [RPSH_BACSU]	11.33	69.15	6.10	65.06	5.74	9.66	0.85	21.48	1.90	22.78	2.01
O34857	Repressor rok OS=Bacillus subtilis (strain 168) GN=rok PE=1 SV=1 - [ROK_BACSU]	0.00	68.58	100.00	38.63	100.00	53.68	100.00	91.97	100.00	77.07	100.00

P94593	Uncharacterized ATP-dependent helicase YwqA OS=Bacillus subtilis (strain 168) GN=ywqA PE=3 SV=2 - [YWQA_BACSU]	0.00	68.42	100.00	50.74	100.00	18.38	100.00	36.35	100.00	55.32	100.00
O34949	Uncharacterized HTH-type transcriptional regulator YkoM OS=Bacillus subtilis (strain 168) GN=ykoM PE=3 SV=1 - [YKOM_BACSU]	13.92	67.52	4.85	37.16	2.67	115.70	8.31	109.26	7.85	122.50	8.80
O34942	ATP-dependent DNA helicase RecG OS=Bacillus subtilis (strain 168) GN=recG PE=3 SV=1 - [RECG_BACSU]	15.59	65.11	4.18	18.82	1.21	8.38	0.54	53.28	3.42	91.14	5.85
O34384	Uncharacterized protein YceE OS=Bacillus subtilis (strain 168) GN=yceE PE=3 SV=1 - [YCEE_BACSU]	25.15	57.11	2.27	42.09	1.67	47.47	1.89	24.93	0.99	28.89	1.15
P54391	Uncharacterized protein YpiF OS=Bacillus subtilis (strain 168) GN=ypiF PE=4 SV=1 - [YPIF_BACSU]	18.13	56.61	3.12	111.48	6.15	34.82	1.92	49.89	2.75	44.21	2.44
P06574	RNA polymerase sigma-B factor OS=Bacillus subtilis (strain 168) GN=sigB PE=1 SV=3 - [RPSB_BACSU]	7.13	55.80	7.82	82.96	11.63	35.84	5.02	43.76	6.14	45.46	6.37
P29141	Minor extracellular protease vpr OS=Bacillus subtilis (strain 168) GN=vpr PE=1 SV=1 - [SUBV_BACSU]	1.89	54.08	28.67	21.77	11.54	58.83	31.18	123.06	65.28	92.17	48.85
P54616	Enoyl-[acyl-carrier-protein] reductase [NADH] FabI OS=Bacillus subtilis (strain 168) GN=fabI PE=1 SV=2 - [FABI_BACSU]	23.49	53.75	2.29	26.57	1.13	63.25	2.69	21.22	0.90	40.63	1.73
P17922	Phenylalanine--tRNA ligase beta subunit OS=Bacillus subtilis (strain 168) GN=pheT PE=3 SV=2 - [SYFB_BACSU]	16.28	49.85	3.06	14.87	0.91	105.73	6.50	12.43	0.76	9.37	0.58
P94461	Primosomal protein N' OS=Bacillus subtilis (strain 168) GN=priA PE=3 SV=2 - [PRIA_BACSU]	0.00	49.85	100.00	3.58	100.00	26.33	100.00	25.73	100.00	57.50	100.00
O05521	Redox-sensing transcriptional repressor Rex OS=Bacillus subtilis (strain 168) GN=rex PE=1 SV=1 - [REX_BACSU]	8.57	49.24	5.74	12.80	1.49	50.05	5.84	59.25	6.91	57.28	6.68
O34526	Alanine--tRNA ligase OS=Bacillus subtilis (strain 168) GN=alaS PE=3 SV=1 - [SYA_BACSU]	21.39	49.16	2.30	11.17	0.52	99.48	4.65	22.60	1.06	34.41	1.61
Q04778	HTH-type transcriptional regulator AlsR OS=Bacillus subtilis (strain 168) GN=alsR PE=3 SV=1 - [ALSR_BACSU]	0.00	47.16	100.00	9.92	100.00	36.67	100.00	14.55	100.00	12.76	100.00
O34303	Type-2 restriction enzyme BsuMI component YdjA OS=Bacillus subtilis (strain 168) GN=ydjA PE=2 SV=1 - [YDJA_BACSU]	0.00	46.79	100.00	20.22	100.00	22.72	100.00	104.27	100.00	92.33	100.00
O34921	Uncharacterized protein YtoI OS=Bacillus subtilis (strain 168) GN=ytoI PE=3 SV=1 - [YTOI_BACSU]	9.40	45.20	4.81	17.61	1.87	45.88	4.88	90.35	9.61	118.09	12.56
P94463	Methionyl-tRNA formyltransferase OS=Bacillus subtilis (strain 168) GN=fmt PE=3 SV=2 - [FMT_BACSU]	15.82	45.07	2.85	8.46	0.53	37.36	2.36	9.46	0.60	2.18	0.14
O31718	UPF0356 protein YkzG OS=Bacillus subtilis (strain 168) GN=ykzG PE=3 SV=1 - [YKZG_BACSU]	0.00	44.63	100.00	56.89	100.00	7.89	100.00	7.64	100.00	10.03	100.00
P96714	Uncharacterized protein YwqB OS=Bacillus subtilis (strain 168) GN=ywqB PE=3 SV=2 - [YWQB_BACSU]	8.88	43.12	4.86	75.35	8.49	0.00	0.00	77.13	8.69	45.42	5.12
Q45498	UPF0637 protein YktB OS=Bacillus subtilis (strain 168) GN=yktB PE=1 SV=1 - [YKTB_BACSU]	3.02	42.04	13.91	29.80	9.86	50.06	16.57	35.54	11.77	39.74	13.15
P71021	Septum site-determining protein DivIVA OS=Bacillus subtilis (strain 168) GN=divIVA PE=1 SV=1 - [DIV4A_BACSU]	12.05	41.86	3.47	55.10	4.57	56.24	4.67	37.87	3.14	40.92	3.40
O35025	Type-2 restriction enzyme BsuMI component YdiR OS=Bacillus subtilis (strain 168) GN=ydiR PE=2 SV=1 - [YDIR_BACSU]	0.00	41.85	100.00	8.88	100.00	78.47	100.00	116.49	100.00	81.94	100.00
P55873	50S ribosomal protein L20 OS=Bacillus subtilis (strain 168) GN=rplT PE=1 SV=1 - [RL20_BACSU]	17.81	41.59	2.34	83.35	4.68	124.51	6.99	118.06	6.63	118.99	6.68
O06974	Gluconeogenesis factor OS=Bacillus subtilis (strain 168) GN=mgfK PE=3 SV=1 - [GNGF_BACSU]	20.20	41.18	2.04	51.36	2.54	36.83	1.82	51.30	2.54	56.75	2.81
O32236	HTH-type transcriptional repressor RghR OS=Bacillus subtilis (strain 168) GN=rghR PE=1 SV=1 - [RGHR_BACSU]	0.00	40.25	100.00	18.08	100.00	21.13	100.00	13.32	100.00	30.78	100.00
P23477	ATP-dependent helicase/deoxyribonuclease subunit B OS=Bacillus subtilis (strain 168) GN=addB PE=1 SV=2 - [ADDB_BACSU]	0.00	39.24	100.00	6.26	100.00	35.65	100.00	18.81	100.00	37.11	100.00
P13792	Alkaline phosphatase synthesis transcriptional regulatory protein PhoP OS=Bacillus subtilis (strain 168) GN=phoP PE=1 SV=4 - [PHOP_BACSU]	14.25	38.91	2.73	22.33	1.57	20.32	1.43	26.30	1.85	33.34	2.34
P37524	Nucleoid occlusion protein OS=Bacillus subtilis (strain 168) GN=noc PE=1 SV=1 - [NOC_BACSU]	14.86	38.44	2.59	26.73	1.80	41.24	2.77	15.41	1.04	30.22	2.03
P37940	2-oxoisovalerate dehydrogenase subunit alpha OS=Bacillus subtilis (strain 168) GN=bfmBAA PE=1 SV=1 - [ODBA_BACSU]	13.00	37.36	2.87	32.41	2.49	90.69	6.98	62.09	4.78	74.58	5.74
P54476	Probable endonuclease 4 OS=Bacillus subtilis (strain 168) GN=nfo PE=3 SV=1 - [END4_BACSU]	10.21	36.86	3.61	31.77	3.11	34.92	3.42	52.58	5.15	46.66	4.57
P37599	Chemotaxis protein CheV OS=Bacillus subtilis (strain 168) GN=cheV PE=1 SV=1 - [CHEV_BACSU]	10.54	36.19	3.43	9.18	0.87	44.32	4.20	36.86	3.50	20.50	1.94
P39601	Uncharacterized HTH-type transcriptional regulator YwcC OS=Bacillus subtilis (strain 168) GN=ywcC PE=3 SV=2 - [YWCC_BACSU]	13.40	35.00	2.61	42.11	3.14	16.88	1.26	37.55	2.80	27.69	2.07
A3F320	Transcriptional regulator and biotin acetyl-CoA-carboxylase synthetase (Fragment) OS=Bacillus subtilis (strain 168) GN=birA PE=4 SV=1 - [A3F320_BACSU]	4.45	34.96	7.86	7.68	1.73	24.07	5.41	37.16	8.36	39.60	8.90
O32264	Probable 2-ketogluconate reductase OS=Bacillus subtilis (strain 168) GN=yvcT PE=3 SV=1 - [TKRA_BACSU]	16.52	33.71	2.04	11.39	0.69	58.56	3.55	31.12	1.88	23.70	1.44
P10726	RNA polymerase sigma-D factor OS=Bacillus subtilis (strain 168) GN=sigD PE=1 SV=2 - [RPSD_BACSU]	0.00	32.92	100.00	9.73	100.00	6.68	100.00	4.41	100.00	3.98	100.00
P24139	Oligopeptide transport system permease protein OppC OS=Bacillus subtilis (strain 168) GN=oppC PE=2 SV=1 - [OPPC_BACSU]	11.69	32.80	2.81	43.39	3.71	10.39	0.89	19.52	1.67	19.03	1.63
O34635	Probable L-serine dehydratase, beta chain OS=Bacillus subtilis (strain 168) GN=sdaAB PE=3 SV=1 - [SDHAB_BACSU]	2.89	32.22	11.17	17.82	6.18	21.77	7.55	21.54	7.47	27.75	9.62
O34484	Methionine aminopeptidase 2 OS=Bacillus subtilis (strain 168) GN=mapB PE=1 SV=1 - [MAP12_BACSU]	13.42	32.11	2.39	30.17	2.25	34.02	2.54	26.10	1.95	26.33	1.96
P40762	Uncharacterized HTH-type transcriptional regulator YvmB OS=Bacillus subtilis (strain 168) GN=yvmB PE=3 SV=1 - [YVMB_BACSU]	2.18	30.97	14.21	18.18	8.34	10.23	4.69	17.77	8.15	36.13	16.57

P39646	Phosphate acetyltransferase OS=Bacillus subtilis (strain 168) GN=pta PE=1 SV=3 - [PTAS_BACSU]	13.64	30.95	2.27	17.54	1.29	103.29	7.57	21.88	1.60	12.45	0.91
P39118	1,4-alpha-glucan branching enzyme GlgB OS=Bacillus subtilis (strain 168) GN=glgB PE=2 SV=1 - [GLGB_BACSU]	9.07	30.80	3.40	24.62	2.72	11.06	1.22	52.17	5.75	42.29	4.67
P39776	Tyrosine recombinase XerC OS=Bacillus subtilis (strain 168) GN=xerC PE=1 SV=1 - [XERC_BACSU]	6.34	29.94	4.72	12.50	1.97	9.90	1.56	37.02	5.84	55.47	8.75
P39586	Uncharacterized protein YwbC OS=Bacillus subtilis (strain 168) GN=ywbC PE=3 SV=1 - [YWBC_BACSU]	7.80	29.90	3.83	53.85	6.90	9.20	1.18	13.22	1.69	12.60	1.61
P39845	Plipastatin synthase subunit A OS=Bacillus subtilis (strain 168) GN=ppsA PE=1 SV=2 - [PPSA_BACSU]	13.00	29.83	2.29	47.59	3.66	47.74	3.67	16.36	1.26	25.82	1.99
P39610	Pyridoxine kinase OS=Bacillus subtilis (strain 168) GN=pdxK PE=1 SV=1 - [PDXK_BACSU]	13.54	29.30	2.16	7.84	0.58	32.55	2.40	0.00	0.00	19.12	1.41
Q795R8	Uncharacterized protein YtfP OS=Bacillus subtilis (strain 168) GN=ytfP PE=4 SV=2 - [YTFP_BACSU]	8.64	29.28	3.39	54.84	6.34	14.84	1.72	22.74	2.63	18.68	2.16
P81102	Putative NAD(P)H nitroreductase YodC OS=Bacillus subtilis (strain 168) GN=yodC PE=1 SV=3 - [YODC_BACSU]	3.72	29.23	7.87	14.04	3.78	16.99	4.57	19.10	5.14	26.10	7.02
P39914	Uncharacterized protein YtxJ OS=Bacillus subtilis (strain 168) GN=ytxJ PE=4 SV=1 - [YTXJ_BACSU]	2.16	28.77	13.33	7.55	3.50	9.62	4.46	8.03	3.72	3.80	1.76
P80240	Transcription elongation factor GreA OS=Bacillus subtilis (strain 168) GN=greA PE=1 SV=4 - [GREA_BACSU]	11.23	28.68	2.55	33.85	3.01	20.92	1.86	17.06	1.52	19.18	1.71
P14802	Uncharacterized oxidoreductase YoxD OS=Bacillus subtilis (strain 168) GN=yoxD PE=3 SV=2 - [YOXD_BACSU]	8.33	27.69	3.32	35.18	4.22	59.39	7.13	22.87	2.75	32.76	3.93
P54452	Uncharacterized protein YqeG OS=Bacillus subtilis (strain 168) GN=yqeG PE=4 SV=1 - [YQEG_BACSU]	11.15	27.51	2.47	63.11	5.66	20.05	1.80	19.61	1.76	19.16	1.72
P24219	RNA polymerase sigma-54 factor OS=Bacillus subtilis (strain 168) GN=sigL PE=3 SV=1 - [RP54_BACSU]	3.66	27.35	7.48	69.67	19.05	0.00	0.00	13.89	3.80	7.18	1.96
P40737	Antitoxin YxxD OS=Bacillus subtilis (strain 168) GN=yxxD PE=1 SV=1 - [YXXD_BACSU]	7.37	26.91	3.65	20.00	2.71	21.39	2.90	12.51	1.70	4.07	0.55
P96582	HTH-type transcriptional regulator LrpC OS=Bacillus subtilis (strain 168) GN=lrpC PE=1 SV=2 - [LRPC_BACSU]	7.15	26.38	3.69	26.37	3.69	45.27	6.33	48.10	6.73	57.03	7.98
P94443	Negative transcription regulator PadR OS=Bacillus subtilis (strain 168) GN=padR PE=4 SV=1 - [PADR_BACSU]	0.00	25.86	100.00	2.35	100.00	11.61	100.00	15.29	100.00	23.70	100.00
O31796	RNA-binding protein Hfq OS=Bacillus subtilis (strain 168) GN=hfq PE=1 SV=1 - [HFQ_BACSU]	7.90	25.35	3.21	9.08	1.15	40.33	5.11	22.98	2.91	27.70	3.51
P94544	DNA polymerase/3'-5' exonuclease PolX OS=Bacillus subtilis (strain 168) GN=polX PE=1 SV=1 - [POLX_BACSU]	3.86	24.74	6.41	9.00	2.33	55.41	14.35	68.55	17.76	111.78	28.95
P54717	HTH-type transcriptional regulator GlvR OS=Bacillus subtilis (strain 168) GN=glvR PE=2 SV=1 - [GLVR_BACSU]	1.73	23.78	13.78	2.48	1.44	2.39	1.39	22.60	13.10	18.93	10.97
Q45065	Uncharacterized protein YneT OS=Bacillus subtilis (strain 168) GN=yneT PE=4 SV=1 - [YNET_BACSU]	9.64	23.19	2.40	7.26	0.75	26.16	2.71	14.16	1.47	12.12	1.26
P71047	Putative HTH-type transcriptional regulator YwgB OS=Bacillus subtilis (strain 168) GN=ywgB PE=3 SV=1 - [YWGB_BACSU]	9.98	22.88	2.29	13.55	1.36	15.35	1.54	10.23	1.02	18.31	1.83
O34827	Uncharacterized HTH-type transcriptional regulator YkuM OS=Bacillus subtilis (strain 168) GN=ykuM PE=3 SV=1 - [YKUM_BACSU]	1.85	22.77	12.34	5.06	2.74	30.91	16.74	22.25	12.05	35.81	19.40
O07001	Uncharacterized HTH-type transcriptional regulator YvdT OS=Bacillus subtilis (strain 168) GN=yvdT PE=1 SV=1 - [YVDT_BACSU]	10.52	22.26	2.11	10.87	1.03	19.61	1.86	20.82	1.98	18.96	1.80
O31648	Uncharacterized N-acetyltransferase YjdG OS=Bacillus subtilis (strain 168) GN=yjdG PE=3 SV=1 - [YJDG_BACSU]	7.63	22.18	2.91	43.38	5.69	0.00	0.00	7.14	0.94	6.57	0.86
O34357	Thioredoxin-like protein YtpP OS=Bacillus subtilis (strain 168) GN=ytpP PE=2 SV=1 - [YTPP_BACSU]	2.68	22.07	8.25	5.53	2.07	17.39	6.50	15.99	5.97	18.39	6.87
P80871	General stress protein 14 OS=Bacillus subtilis (strain 168) GN=ywrO PE=1 SV=2 - [GS14_BACSU]	5.29	21.80	4.12	3.76	0.71	22.53	4.26	9.05	1.71	7.28	1.38
P36843	Arginine biosynthesis bifunctional protein ArgJ OS=Bacillus subtilis (strain 168) GN=argJ PE=3 SV=2 - [ARGJ_BACSU]	7.87	21.55	2.74	10.08	1.28	15.62	1.98	0.00	0.00	18.34	2.33
O34752	Prolipoprotein diacylglyceryl transferase OS=Bacillus subtilis (strain 168) GN=lgt PE=1 SV=1 - [LGT_BACSU]	0.00	21.44	100.00	22.72	100.00	3.72	100.00	19.51	100.00	6.84	100.00
P32395	Uroporphyrinogen decarboxylase OS=Bacillus subtilis (strain 168) GN=hemE PE=1 SV=1 - [DCUP_BACSU]	9.21	21.10	2.29	22.66	2.46	36.19	3.93	14.67	1.59	14.65	1.59
O34948	Uncharacterized oxidoreductase YkwC OS=Bacillus subtilis (strain 168) GN=ykwC PE=3 SV=1 - [YKWC_BACSU]	9.77	21.05	2.15	15.47	1.58	32.09	3.28	10.82	1.11	25.68	2.63
P39156	Putative sugar phosphate isomerase YwlF OS=Bacillus subtilis (strain 168) GN=ywlF PE=2 SV=1 - [YWLF_BACSU]	1.98	20.41	10.33	5.84	2.96	24.79	12.55	0.00	0.00	3.46	1.75
O32078	Uncharacterized protein YuaE OS=Bacillus subtilis (strain 168) GN=yuaE PE=4 SV=1 - [YUAE_BACSU]	8.26	20.16	2.44	47.65	5.77	56.01	6.78	40.78	4.94	22.92	2.78
P54390	UPF0302 protein YpiB OS=Bacillus subtilis (strain 168) GN=ypiB PE=3 SV=1 - [YPIB_BACSU]	2.13	19.75	9.27	2.81	1.32	15.03	7.05	12.76	5.99	4.42	2.07
O06724	Uncharacterized protein YisK OS=Bacillus subtilis (strain 168) GN=yisK PE=2 SV=1 - [YISK_BACSU]	5.92	19.48	3.29	13.10	2.21	47.90	8.09	14.44	2.44	13.69	2.31
P46354	Purine nucleoside phosphorylase 1 OS=Bacillus subtilis (strain 168) GN=punA PE=1 SV=1 - [PUNA_BACSU]	5.53	19.36	3.50	15.42	2.79	38.11	6.89	16.10	2.91	13.53	2.45
Q45499	Inositol-1-monophosphatase OS=Bacillus subtilis (strain 168) GN=suhB PE=3 SV=1 - [SUHB_BACSU]	1.89	19.35	10.24	4.43	2.34	18.30	9.68	2.07	1.10	6.57	3.47
P94363	Citrate/malate transporter OS=Bacillus subtilis (strain 168) GN=cimH PE=1 SV=1 - [CIMH_BACSU]	6.49	19.31	2.97	7.33	1.13	13.63	2.10	10.44	1.61	19.67	3.03
P37954	UvrABC system protein B OS=Bacillus subtilis (strain 168) GN=uvrB PE=1 SV=2 - [UVRB_BACSU]	1.61	18.92	11.75	7.25	4.50	0.00	0.00	0.00	0.00	3.62	2.25
P96628	Protein SprT-like OS=Bacillus subtilis (strain 168) GN=ydcK PE=3 SV=1 - [SPRTL_BACSU]	7.73	18.65	2.41	27.12	3.51	2.63	0.34	29.11	3.77	23.24	3.01
O31593	Putative efflux system component YhbJ OS=Bacillus subtilis (strain 168) GN=yhbJ PE=3 SV=1 - [YHBJ_BACSU]	7.03	18.39	2.61	5.43	0.77	18.29	2.60	10.80	1.54	3.72	0.53

P39066	Acetoin utilization protein AcuB OS=Bacillus subtilis (strain 168) GN=acuB PE=3 SV=1 - [ACUB_BACSU]	2.65	18.25	6.87	28.04	10.56	14.55	5.48	6.93	2.61	6.05	2.28
P96608	Putative acyl-CoA dehydrogenase YdbM OS=Bacillus subtilis (strain 168) GN=ydbM PE=2 SV=1 - [YDBM_BACSU]	5.10	17.97	3.53	6.44	1.26	36.86	7.23	8.38	1.64	17.45	3.42
O32248	Uncharacterized N-acetyltransferase YvbK OS=Bacillus subtilis (strain 168) GN=yvbK PE=1 SV=1 - [YVBK_BACSU]	8.32	17.81	2.14	19.87	2.39	25.17	3.02	11.55	1.39	19.26	2.31
P32727	Transcription termination/antitermination protein NusA OS=Bacillus subtilis (strain 168) GN=nusA PE=3 SV=2 - [NUSA_BACSU]	6.09	17.63	2.90	10.04	1.65	41.19	6.77	21.03	3.46	25.92	4.26
P71036	Putative HTH-type transcriptional regulator YwnA OS=Bacillus subtilis (strain 168) GN=ywnA PE=1 SV=1 - [YWNA_BACSU]	1.82	17.57	9.67	4.02	2.21	14.46	7.96	14.65	8.07	24.42	13.45
O31737	Uncharacterized protein YlqB OS=Bacillus subtilis (strain 168) GN=ylqB PE=1 SV=1 - [YLQB_BACSU]	0.00	17.54	100.00	14.26	100.00	3.31	100.00	9.74	100.00	7.47	100.00
Q45599	Uncharacterized protein YydC OS=Bacillus subtilis (strain 168) GN=yydC PE=4 SV=1 - [YYDC_BACSU]	3.17	17.52	5.53	5.32	1.68	13.07	4.13	13.83	4.37	11.63	3.67
Q45549	Transcriptional repressor NrdR OS=Bacillus subtilis (strain 168) GN=nrdR PE=3 SV=2 - [NRDR_BACSU]	8.18	16.89	2.06	20.01	2.44	17.50	2.14	16.27	1.99	19.31	2.36
O34305	Uncharacterized protein YtoQ OS=Bacillus subtilis (strain 168) GN=ytoQ PE=4 SV=1 - [YTOQ_BACSU]	3.65	16.71	4.57	5.33	1.46	14.50	3.97	6.20	1.70	8.08	2.21
O07939	Uncharacterized protein YisT OS=Bacillus subtilis (strain 168) GN=yisT PE=3 SV=1 - [YIST_BACSU]	0.00	16.42	100.00	34.42	100.00	10.28	100.00	8.98	100.00	2.98	100.00
O34841	Uncharacterized protein YoeB OS=Bacillus subtilis (strain 168) GN=yoeB PE=1 SV=2 - [YOEB_BACSU]	6.85	16.32	2.38	11.48	1.68	8.38	1.22	16.08	2.35	7.59	1.11
P54159	Uncharacterized protein YpbR OS=Bacillus subtilis (strain 168) GN=ypbR PE=4 SV=1 - [YPBR_BACSU]	5.07	15.80	3.11	5.76	1.14	11.61	2.29	8.37	1.65	0.00	0.00
O07636	Uncharacterized protein YlaL OS=Bacillus subtilis (strain 168) GN=ylaL PE=4 SV=1 - [YLAL_BACSU]	0.00	15.73	100.00	13.61	100.00	21.21	100.00	5.60	100.00	10.19	100.00
O32126	UPF0331 protein YutE OS=Bacillus subtilis (strain 168) GN=yutE PE=1 SV=1 - [YUTE_BACSU]	7.46	15.67	2.10	7.99	1.07	16.79	2.25	7.77	1.04	3.95	0.53
P54512	Transcriptional regulator MntR OS=Bacillus subtilis (strain 168) GN=mntR PE=1 SV=2 - [MNTR_BACSU]	0.00	15.63	100.00	7.06	100.00	3.09	100.00	3.79	100.00	3.78	100.00
P96642	Uncharacterized protein YddE OS=Bacillus subtilis (strain 168) GN=yddE PE=4 SV=1 - [YDDE_BACSU]	3.32	15.49	4.66	6.89	2.08	9.42	2.84	6.79	2.04	7.23	2.18
P37252	Acetolactate synthase small subunit OS=Bacillus subtilis (strain 168) GN=ilvH PE=3 SV=3 - [ILVH_BACSU]	4.41	15.49	3.51	3.16	0.72	3.46	0.78	6.24	1.41	4.53	1.03
P04948	Homoserine kinase OS=Bacillus subtilis (strain 168) GN=thrB PE=3 SV=2 - [KHSE_BACSU]	2.10	15.41	7.34	9.82	4.68	11.38	5.42	15.00	7.15	5.56	2.65
O34381	HTH-type transcriptional regulator PksA OS=Bacillus subtilis (strain 168) GN=pksA PE=3 SV=1 - [PKSA_BACSU]	0.00	15.38	100.00	39.39	100.00	7.60	100.00	11.15	100.00	11.33	100.00
Q796Y8	Putative peroxiredoxin YgaF OS=Bacillus subtilis (strain 168) GN=ygaF PE=3 SV=1 - [BCP_BACSU]	0.00	15.32	100.00	16.39	100.00	21.78	100.00	10.19	100.00	4.94	100.00
O31675	7-cyano-7-deazaguanine synthase OS=Bacillus subtilis (strain 168) GN=queC PE=1 SV=1 - [QUEC_BACSU]	7.50	15.29	2.04	5.69	0.76	8.15	1.09	2.07	0.28	8.49	1.13
P71019	Malonyl CoA-acyl carrier protein transacylase OS=Bacillus subtilis (strain 168) GN=fabD PE=3 SV=2 - [FABD_BACSU]	3.76	15.24	4.05	1.81	0.48	44.93	11.95	5.62	1.50	5.45	1.45
P94588	Uncharacterized protein YwpF OS=Bacillus subtilis (strain 168) GN=ywpF PE=4 SV=1 - [YWPF_BACSU]	5.06	14.90	2.94	27.95	5.52	8.57	1.69	22.82	4.51	13.51	2.67
P94512	Putative uncharacterized hydrolase YsaA OS=Bacillus subtilis (strain 168) GN=ysaA PE=3 SV=2 - [YSAA_BACSU]	4.71	14.63	3.10	0.00	0.00	3.52	0.75	0.00	0.00	2.81	0.60
P50843	4-deoxy-L-threo-5-hexosulose-uronate ketol-isomerase OS=Bacillus subtilis (strain 168) GN=kduI PE=2 SV=1 - [KDUI_BACSU]	5.73	14.41	2.52	0.00	0.00	15.56	2.72	3.46	0.60	0.00	0.00
P71015	HTH-type transcriptional repressor GbsR OS=Bacillus subtilis (strain 168) GN=gbsR PE=3 SV=1 - [GBSR_BACSU]	1.82	14.39	7.90	9.90	5.44	20.25	11.12	16.89	9.27	26.07	14.32
O31504	Putative DNA methyltransferase YeeA OS=Bacillus subtilis (strain 168) GN=yeeA PE=4 SV=1 - [YEEA_BACSU]	0.00	14.20	100.00	4.03	100.00	15.68	100.00	12.29	100.00	35.45	100.00
O31727	UPF0001 protein YlmE OS=Bacillus subtilis (strain 168) GN=ylmE PE=3 SV=1 - [YLME_BACSU]	5.99	14.15	2.36	7.95	1.33	13.86	2.31	0.00	0.00	12.31	2.05
P42976	4-hydroxy-tetrahydrodipicolinate reductase OS=Bacillus subtilis (strain 168) GN=dapB PE=3 SV=2 - [DAPB_BACSU]	6.82	14.01	2.05	7.77	1.14	28.62	4.20	1.78	0.26	11.28	1.65
P45943	Response regulator aspartate phosphatase E OS=Bacillus subtilis (strain 168) GN=rapE PE=3 SV=2 - [RAPE_BACSU]	4.93	13.60	2.76	12.10	2.45	0.00	0.00	35.22	7.15	14.93	3.03
P94559	Putative metallophosphoesterase YsnB OS=Bacillus subtilis (strain 168) GN=ysnB PE=3 SV=2 - [YSNB_BACSU]	0.00	13.59	100.00	22.77	100.00	10.83	100.00	6.93	100.00	10.09	100.00
P55340	Protein EcsB OS=Bacillus subtilis (strain 168) GN=ecsB PE=4 SV=1 - [ECSB_BACSU]	3.88	13.39	3.45	18.57	4.78	0.00	0.00	6.77	1.74	12.48	3.21
O34970	Probable HTH-type transcriptional regulator YttP OS=Bacillus subtilis (strain 168) GN=yttP PE=2 SV=1 - [YTTP_BACSU]	3.75	13.31	3.55	30.70	8.20	0.00	0.00	8.65	2.31	5.70	1.52
P49778	Elongation factor P OS=Bacillus subtilis (strain 168) GN=efp PE=3 SV=2 - [EFP_BACSU]	0.00	13.06	100.00	9.52	100.00	4.51	100.00	9.97	100.00	10.26	100.00
P32730	Uncharacterized protein YlxP OS=Bacillus subtilis (strain 168) GN=ylxP PE=4 SV=1 - [YLXP_BACSU]	0.00	12.97	100.00	13.69	100.00	10.43	100.00	11.04	100.00	10.89	100.00
O34592	AB hydrolase superfamily protein YdjP OS=Bacillus subtilis (strain 168) GN=ydjP PE=2 SV=1 - [YDJP_BACSU]	6.06	12.85	2.12	11.85	1.95	10.97	1.81	2.37	0.39	6.86	1.13
P06567	Primosomal protein DnaI OS=Bacillus subtilis (strain 168) GN=dnaI PE=1 SV=1 - [DNAI_BACSU]	5.14	12.67	2.46	2.26	0.44	0.00	0.00	4.41	0.86	0.00	0.00
P94359	Uncharacterized protein YxkF OS=Bacillus subtilis (strain 168) GN=yxkF PE=4 SV=1 - [YXKF_BACSU]	0.00	12.66	100.00	0.00	100.00	23.04	100.00	13.43	100.00	5.00	100.00
O32044	Single-stranded-DNA-specific exonuclease RecJ OS=Bacillus subtilis (strain 168) GN=recJ PE=3 SV=1 - [RECJ_BACSU]	3.68	12.63	3.43	0.00	0.00	11.59	3.15	5.35	1.45	10.42	2.83
P39694	ComE operon protein 1 OS=Bacillus subtilis (strain 168) GN=comEA PE=1 SV=1 - [COMEA_BACSU]	0.00	12.26	100.00	14.18	100.00	5.56	100.00	25.19	100.00	15.41	100.00

O07624	Uncharacterized beta-barrel protein YwiB OS=Bacillus subtilis (strain 168) GN=ywiB PE=1 SV=1 - [YWIB_BACSU]	0.00	11.98	100.00	8.62	100.00	2.17	100.00	10.26	100.00	6.98	100.00
O34403	Formamidopyrimidine-DNA glycosylase OS=Bacillus subtilis (strain 168) GN=mutM PE=3 SV=4 - [FPG_BACSU]	3.08	11.98	3.88	0.00	0.00	8.99	2.92	10.83	3.51	18.48	5.99
O32253	Central glycolytic genes regulator OS=Bacillus subtilis (strain 168) GN=cggR PE=1 SV=1 - [CGGR_BACSU]	1.66	11.78	7.08	2.35	1.41	29.48	17.72	38.68	23.25	37.74	22.68
P54574	Ferric uptake regulation protein OS=Bacillus subtilis (strain 168) GN=fur PE=1 SV=2 - [FUR_BACSU]	1.90	11.75	6.20	8.91	4.70	10.00	5.27	7.37	3.89	4.74	2.50
P42961	Uncharacterized protein YcsD OS=Bacillus subtilis (strain 168) GN=ycsD PE=3 SV=2 - [YCSD_BACSU]	0.00	11.75	100.00	14.26	100.00	1.81	100.00	3.52	100.00	11.25	100.00
O34527	HTH-type transcriptional regulator CymR OS=Bacillus subtilis (strain 168) GN=cymR PE=1 SV=2 - [CYMR_BACSU]	2.39	11.59	4.85	5.30	2.22	21.35	8.94	12.05	5.04	10.36	4.34
O34331	Putative rRNA methyltransferase YlbH OS=Bacillus subtilis (strain 168) GN=ylbH PE=3 SV=2 - [YLBH_BACSU]	3.20	11.43	3.57	39.99	12.49	24.54	7.66	13.00	4.06	15.98	4.99
O31494	Uncharacterized HTH-type transcriptional regulator YdzF OS=Bacillus subtilis (strain 168) GN=ydzF PE=3 SV=1 - [YDZF_BACSU]	5.36	11.42	2.13	14.47	2.70	1.96	0.37	10.39	1.94	10.14	1.89
O32006	Resolvase homolog YokA OS=Bacillus subtilis (strain 168) GN=yokA PE=3 SV=1 - [YOKA_BACSU]	3.67	11.29	3.07	3.31	0.90	1.97	0.54	38.65	10.52	24.10	6.56
P37568	Transcriptional regulator CtsR OS=Bacillus subtilis (strain 168) GN=ctsR PE=1 SV=1 - [CTSR_BACSU]	3.97	10.74	2.71	10.81	2.72	4.65	1.17	5.35	1.35	4.82	1.21
P96579	Putative ribosomal N-acetyltransferase YdaF OS=Bacillus subtilis (strain 168) GN=ydaF PE=1 SV=1 - [YDAF_BACSU]	0.00	10.54	100.00	9.46	100.00	18.99	100.00	8.88	100.00	2.30	100.00
O34714	Oxalate decarboxylase OxdC OS=Bacillus subtilis (strain 168) GN=oxdC PE=1 SV=1 - [OXDC_BACSU]	3.71	10.37	2.80	4.88	1.32	6.81	1.84	0.00	0.00	1.93	0.52
P37565	33 kDa chaperonin OS=Bacillus subtilis (strain 168) GN=hslO PE=1 SV=1 - [HSLO_BACSU]	2.75	10.35	3.76	0.00	0.00	9.17	3.34	0.00	0.00	2.03	0.74
P70993	Uncharacterized HTH-type transcriptional regulator YwhA OS=Bacillus subtilis (strain 168) GN=ywhA PE=3 SV=1 - [YWHA_BACSU]	0.00	10.33	100.00	6.43	100.00	23.34	100.00	17.88	100.00	28.87	100.00
O07617	Uncharacterized phosphatase PhoE OS=Bacillus subtilis (strain 168) GN=phoE PE=3 SV=1 - [PHOE_BACSU]	1.86	10.29	5.55	6.34	3.42	14.37	7.75	3.77	2.03	8.29	4.47
P54591	Uncharacterized ABC transporter ATP-binding protein YhcG OS=Bacillus subtilis (strain 168) GN=yhcG PE=3 SV=1 - [YHCG_BACSU]	0.00	10.29	100.00	5.70	100.00	17.01	100.00	11.59	100.00	10.33	100.00
P94352	Uncharacterized protein YxjI OS=Bacillus subtilis (strain 168) GN=yxjI PE=3 SV=1 - [YXJI_BACSU]	3.32	10.24	3.09	13.38	4.04	7.67	2.31	4.82	1.45	6.83	2.06
P81101	Ribosome-recycling factor OS=Bacillus subtilis (strain 168) GN=frr PE=1 SV=2 - [RRF_BACSU]	3.59	10.22	2.85	15.50	4.32	28.96	8.08	6.45	1.80	6.48	1.81
P40400	Putative aliphatic sulfonates-binding protein OS=Bacillus subtilis (strain 168) GN=ssuA PE=2 SV=1 - [SSUA_BACSU]	3.67	10.16	2.77	4.39	1.20	0.00	0.00	0.00	0.00	0.00	0.00
P54389	TPR repeat-containing protein YpiA OS=Bacillus subtilis (strain 168) GN=ypiA PE=3 SV=1 - [YPIA_BACSU]	0.00	10.03	100.00	105.97	100.00	3.82	100.00	1.90	100.00	3.68	100.00
P94548	Fatty acid metabolism regulator protein OS=Bacillus subtilis (strain 168) GN=fadR PE=1 SV=1 - [FADR_BACSU]	1.69	10.03	5.94	8.26	4.90	3.64	2.16	2.54	1.50	4.37	2.59

Sanders et al., Supplementary Figure 1

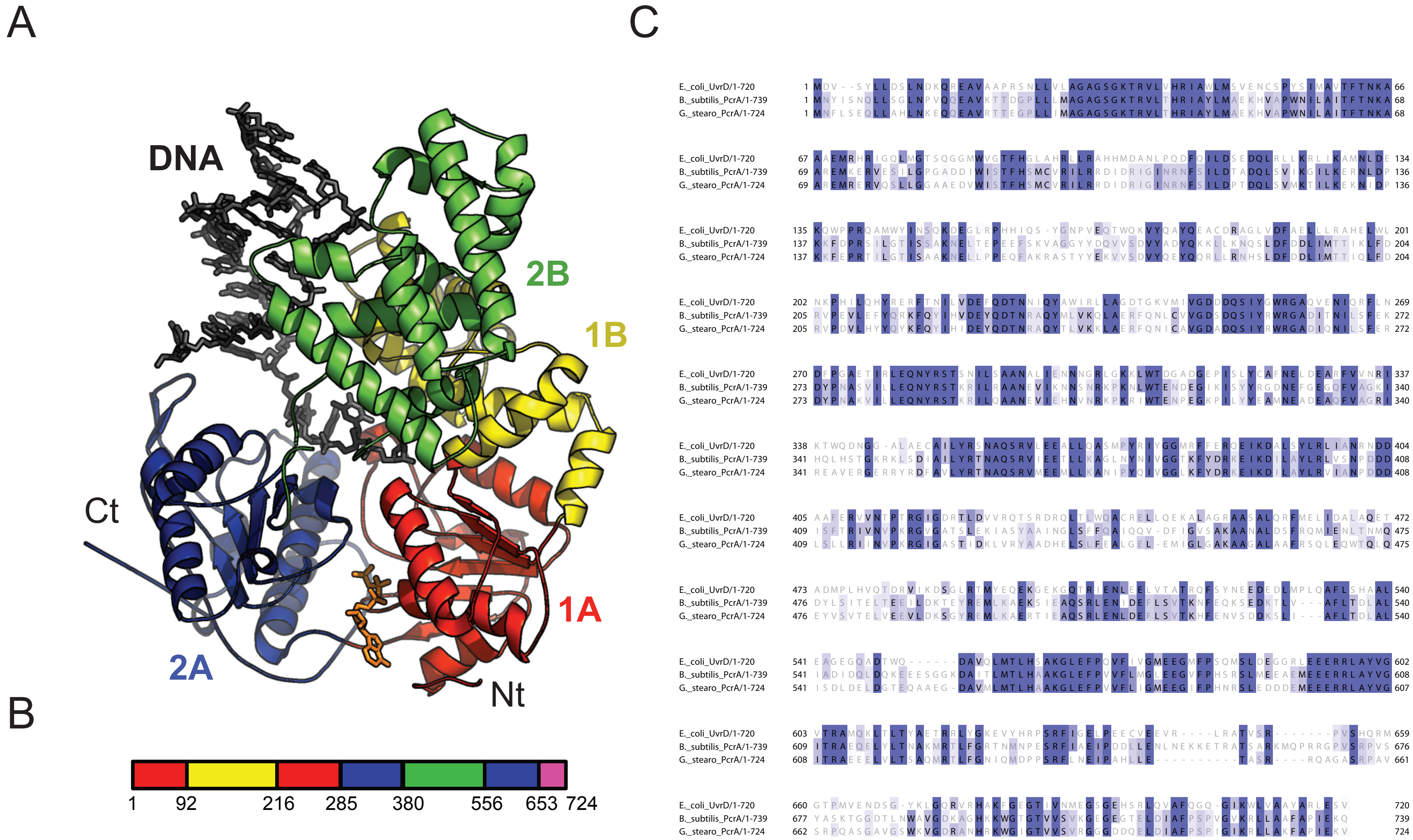

Sanders et al., Supplementary Figure 2

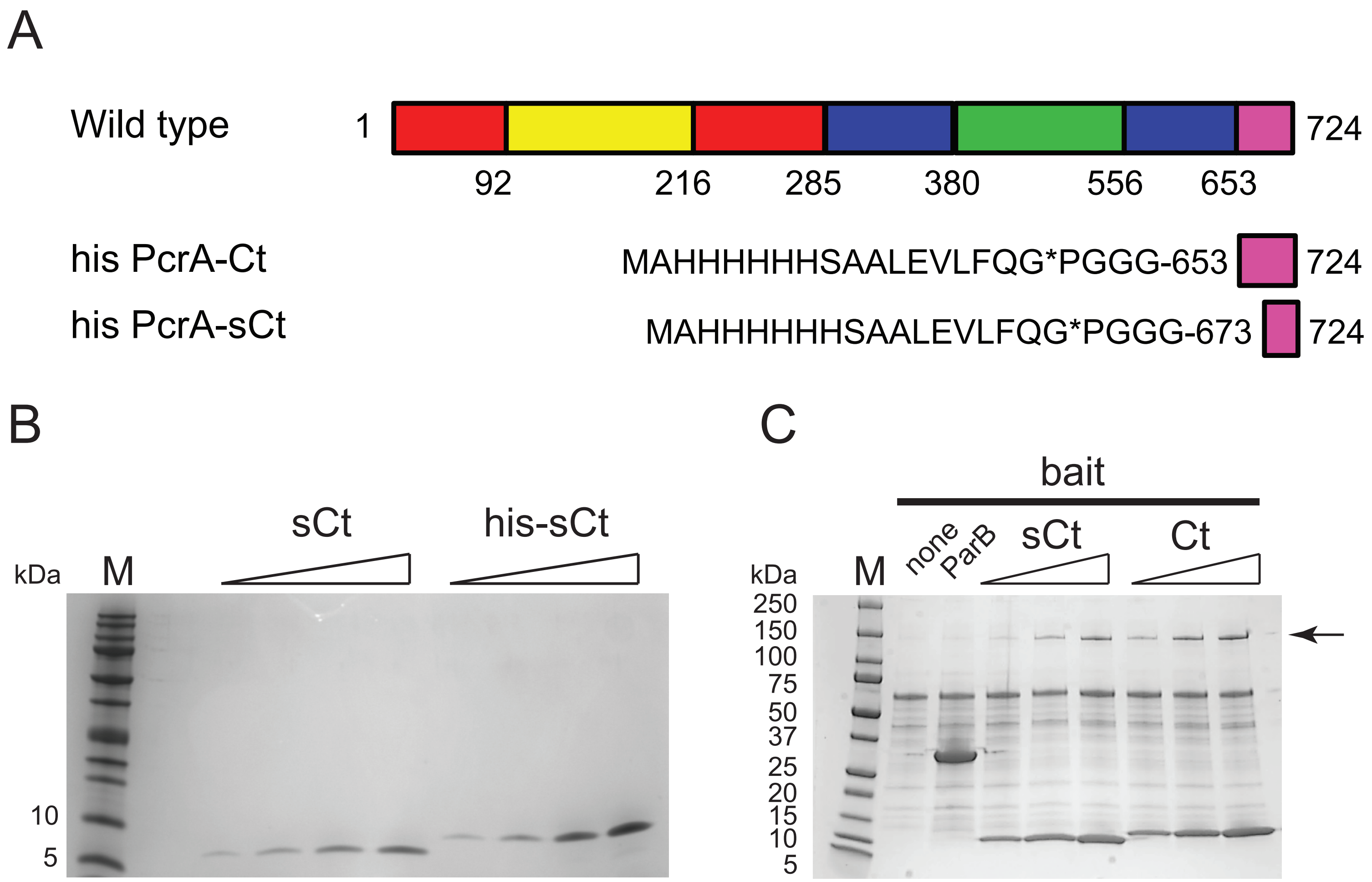

Sanders et al., Supplementary Figure 3

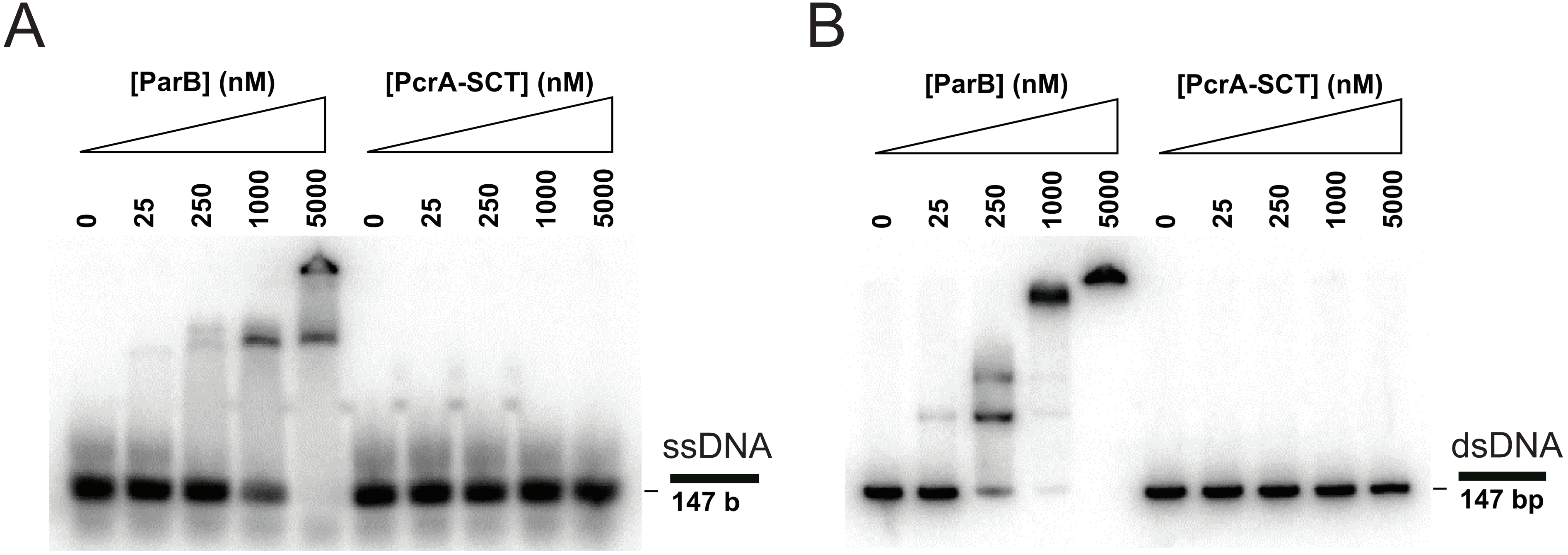

Sanders et al., Supplementary Figure 4

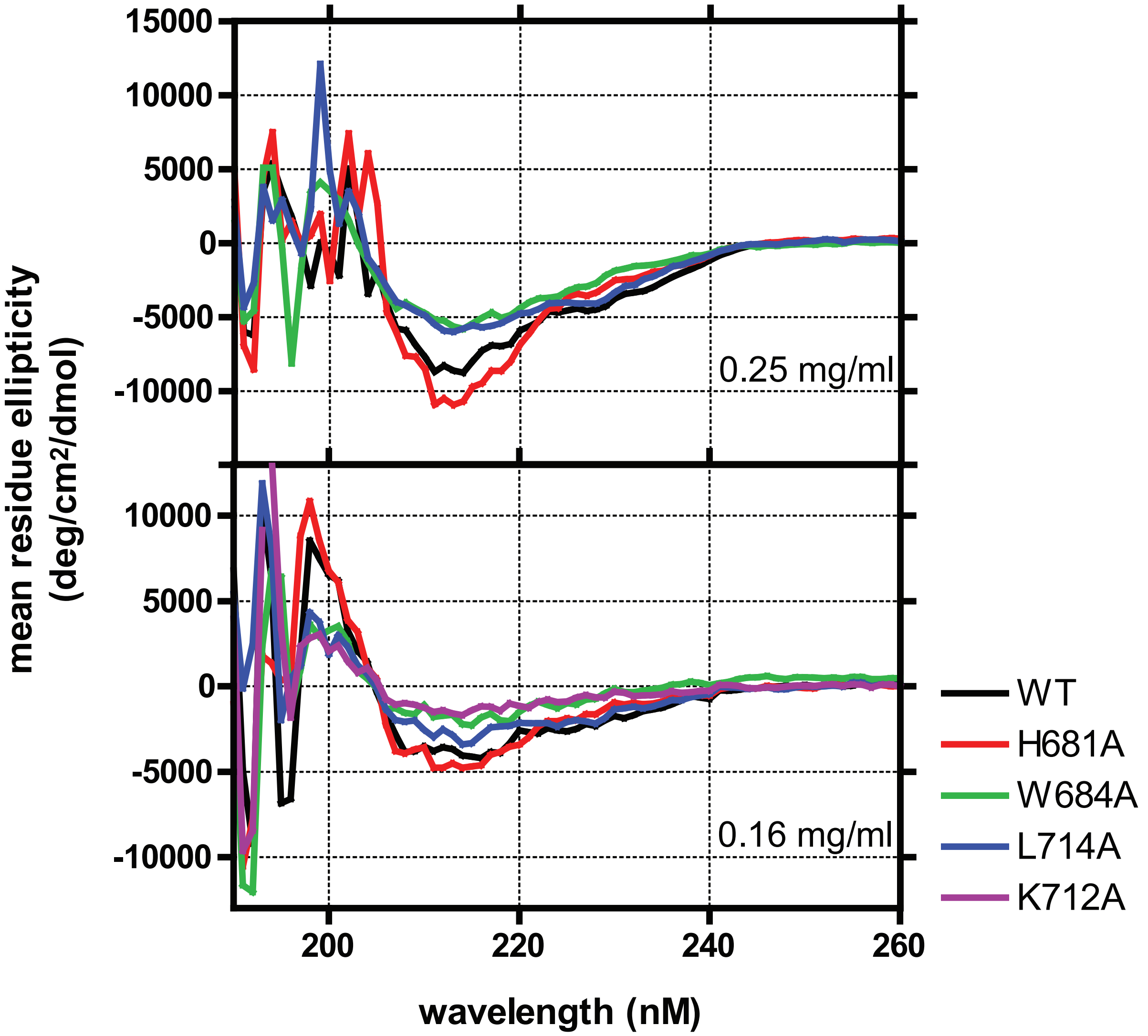

Sanders et al., Supplementary Figure 5

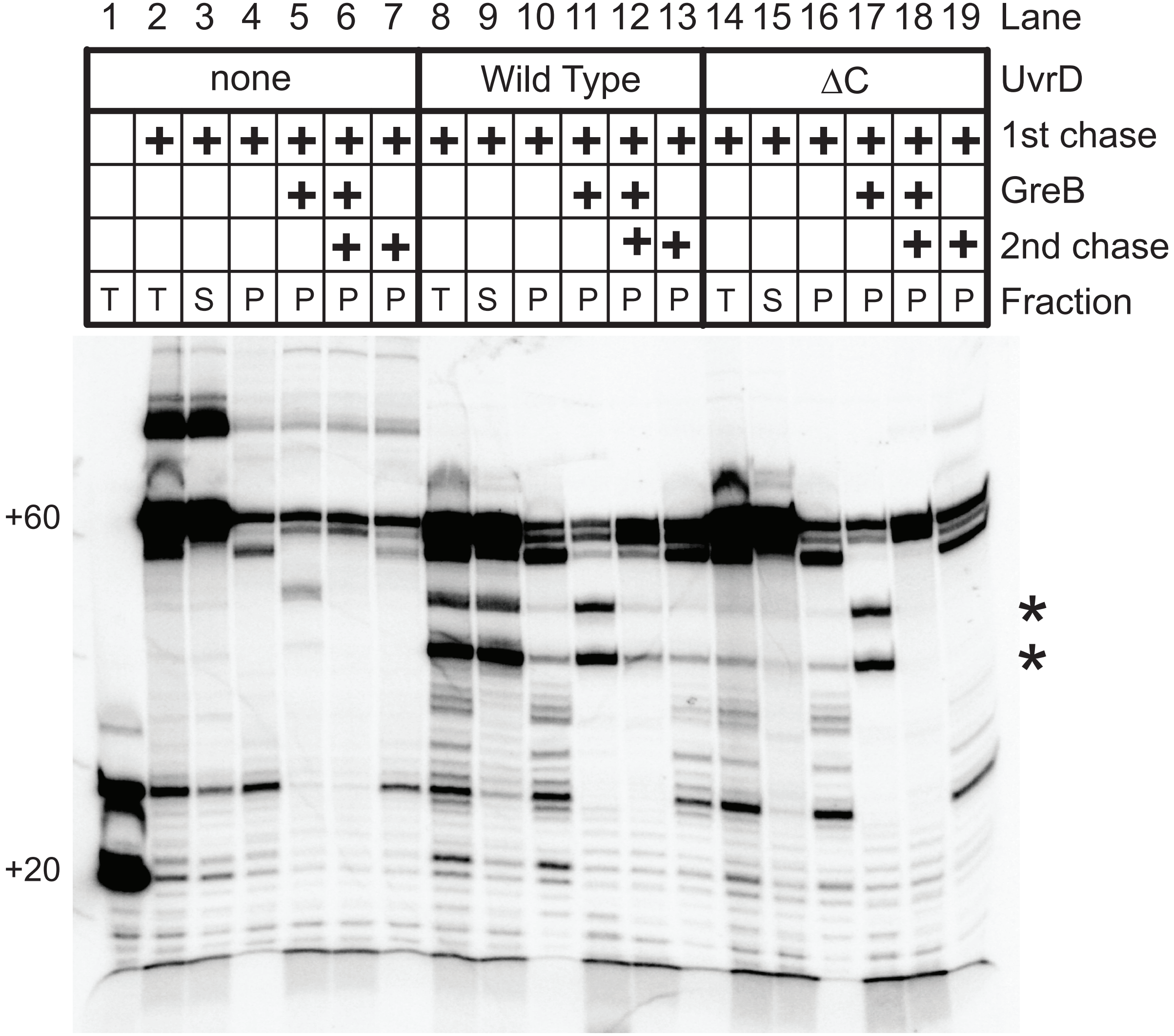

Sanders et al., Supplementary Figure 6

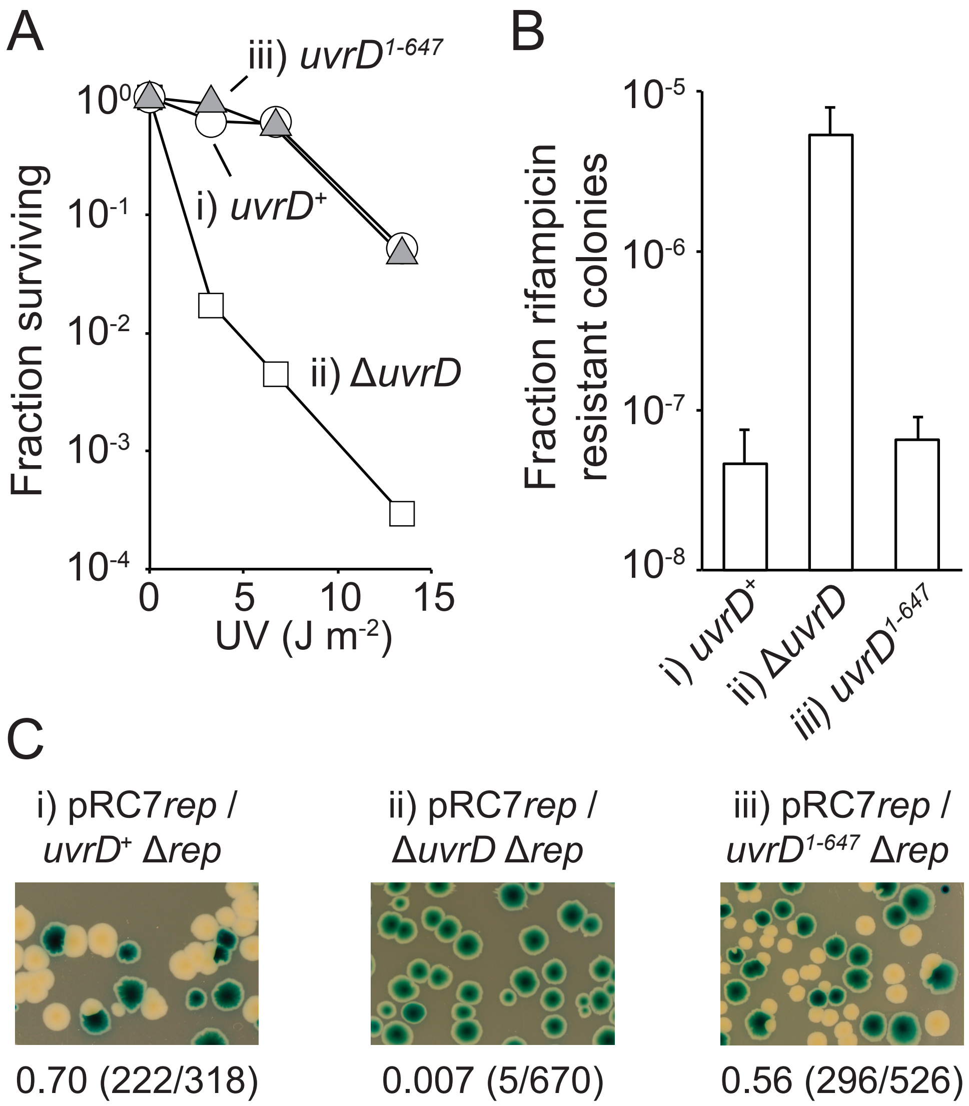

Supplement: Supplementary Data [file gkx074_Supp.pdf]
